# Organization of mouse prefrontal cortex subnetwork revealed by spatial single-cell multi-omic analysis of SPIDER-Seq

**DOI:** 10.1093/nsr/nwag004

**Published:** 2026-01-16

**Authors:** Leqiang Sun, Hu Zheng, Yayu Huang, Xuehuan Huang, Keji Yan, Zhongchao Wang, Liyao Yang, Yiping Yue, Xiaojuan Gou, Guohua Du, Yang Wang, Xiaofeng Wu, Huazhen Liu, Hang Chen, Daqing Ma, Yunyun Han, Jinxia Dai, Gang Cao

**Affiliations:** State Key Laboratory of Agricultural Microbiology, Huazhong Agricultural University, Wuhan 430070, China; College of Veterinary Medicine, Huazhong Agricultural University, Wuhan 430070, China; State Key Laboratory of Agricultural Microbiology, Huazhong Agricultural University, Wuhan 430070, China; College of Veterinary Medicine, Huazhong Agricultural University, Wuhan 430070, China; Faculty of Life and Health Sciences, Shenzhen University of Advanced Technology, Shenzhen 518107, China; Faculty of Life and Health Sciences, and Shenzhen-Hong Kong Institute of Brain Science and The Brain Cognition and Brain Disease Institute, Shenzhen Institute of Advanced Technology, Chinese Academy of Sciences, Shenzhen 518000, China; State Key Laboratory of Agricultural Microbiology, Huazhong Agricultural University, Wuhan 430070, China; College of Veterinary Medicine, Huazhong Agricultural University, Wuhan 430070, China; State Key Laboratory of Agricultural Microbiology, Huazhong Agricultural University, Wuhan 430070, China; College of Veterinary Medicine, Huazhong Agricultural University, Wuhan 430070, China; State Key Laboratory of Agricultural Microbiology, Huazhong Agricultural University, Wuhan 430070, China; College of Veterinary Medicine, Huazhong Agricultural University, Wuhan 430070, China; State Key Laboratory of Agricultural Microbiology, Huazhong Agricultural University, Wuhan 430070, China; College of Veterinary Medicine, Huazhong Agricultural University, Wuhan 430070, China; State Key Laboratory of Agricultural Microbiology, Huazhong Agricultural University, Wuhan 430070, China; College of Life Science and Technology, Huazhong Agriculture University, Wuhan 430070, China; State Key Laboratory of Agricultural Microbiology, Huazhong Agricultural University, Wuhan 430070, China; College of Veterinary Medicine, Huazhong Agricultural University, Wuhan 430070, China; State Key Laboratory of Agricultural Microbiology, Huazhong Agricultural University, Wuhan 430070, China; College of Veterinary Medicine, Huazhong Agricultural University, Wuhan 430070, China; State Key Laboratory of Agricultural Microbiology, Huazhong Agricultural University, Wuhan 430070, China; College of Veterinary Medicine, Huazhong Agricultural University, Wuhan 430070, China; State Key Laboratory of Agricultural Microbiology, Huazhong Agricultural University, Wuhan 430070, China; College of Veterinary Medicine, Huazhong Agricultural University, Wuhan 430070, China; State Key Laboratory of Agricultural Microbiology, Huazhong Agricultural University, Wuhan 430070, China; College of Veterinary Medicine, Huazhong Agricultural University, Wuhan 430070, China; Institute of Highland Forest Science, Chinese Academy of Forestry, Kunming 650224, China; Perioperative and Systems Medicine Laboratory and Department of Anesthesiology, National Clinical Research Center for Child Health; Department of Anesthesiology, Children’s Hospital, Zhejiang University School of Medicine, Hangzhou 310006, China; Division of Anaesthetics, Pain Medicine and Intensive Care, Department of Surgery and Cancer, Faculty of Medicine, Imperial College London, Chelsea & Westminster Hospital, London SW10 9NH, UK; School of Basic Medicine, Tongji Medical College, Huazhong University of Science and Technology, Wuhan 430030, China; State Key Laboratory of Agricultural Microbiology, Huazhong Agricultural University, Wuhan 430070, China; College of Veterinary Medicine, Huazhong Agricultural University, Wuhan 430070, China; Faculty of Life and Health Sciences, Shenzhen University of Advanced Technology, Shenzhen 518107, China; Faculty of Life and Health Sciences, and Shenzhen-Hong Kong Institute of Brain Science and The Brain Cognition and Brain Disease Institute, Shenzhen Institute of Advanced Technology, Chinese Academy of Sciences, Shenzhen 518000, China

**Keywords:** SPIDER-Seq, mouse prefrontal cortex, projectome, transcriptome, spatial-omics

## Abstract

Deciphering the connectome, anatomy, transcriptome and spatial-omics integrated multi-modal brain atlas and its underlying organization principles remains a great challenge. We developed a Single-cell Projectome-transcriptome In situ Deciphering Sequencing (SPIDER-Seq) technique by combining viral barcoding tracing with single-cell sequencing and spatial-omics. This empowers us to delineate an integrated single-cell spatial molecular, cellular, anatomic and projectomic atlas of the mouse prefrontal cortex (PFC). The projectomic and transcriptomic cell clusters display distinct modular organization principles, but are coordinately configured in the PFC. The projection neurons gradiently occupied different territories in the PFC aligning with their wiring patterns. Importantly, they show higher co-projection probability to the downstream nuclei with reciprocal circuit connections. Moreover, we integrated the projectomic atlas with its distinct spectrum of neurotransmitters/neuropeptides with their receptor-related gene profiles in order to demonstrate the PFC neural signal transmission network, by which means we uncovered potential mechanisms underlying the complexity and specificity of neural transmission. Finally, leveraging machine learning, we predicted neuron projections with high accuracy by combining gene profiles and spatial information. As a proof of concept, we used this model to predict projections of fear recall engram neurons. This study facilitates our understanding of the brain multi-modal network and neural computation.

## INTRODUCTION

Deciphering the sophisticated neural connectome and the underlying wiring logic is essential for understanding brain computation and advancing artificial intelligence [1,2]. Although the mesoscopic connectivity in several model organisms have been well characterized [3–7], the detailed wiring information at single neuron resolution remains largely unknown [8,9], impeding the deep understanding of neural computational logic at level of precision. Distinct signaling molecules and wiring-related genes expressed by individual neurons result in their unique and dynamic capacities for information transmission and plasticity. These gene expression profiles, in combination with the neural projection patterns and connection topological organization, make neural networks dynamic and highly integrated complex systems [10]. The integrative analysis of the multi-modal characteristics of these neural network fundamental units with single neuron resolution is essential for understanding the functions of neural networks. Thus, high-throughput neuronal connectivity decoding techniques with the capacity to integrate gene expression and spatial distribution with single cell resolution are highly desirable.

In the past decade, middle- or high-throughput methods for analyzing neuronal connectivity have been developed, such as MAPseq, BARseq, BRICseq and fMOST, revolutionizing the neural network research paradigm and providing new insights into the topological structures of neural circuits [11–15]. BARseq2, an improved version of BARseq, offers a potential path to uncovering the molecular logic underlying neuronal circuits [16]. Because the replication of the barcode-carrying Sindbis virus perturbs neuronal transcription, there is a need for combining a low-toxicity circuit-tracing virus with single-cell sequencing to simultaneously explore circuit architecture and intact transcriptomics. Technologies such as VECTORseq, Retro-Seq and Epi-Retro-Seq have been developed to decipher transcriptomes and even epigenomes in specific circuits [17–20]. However, these methods are insufficient for resolving the complex projection patterns of multiplex projection neurons. Recently MERGE-Seq and Projection-seq, which use a low-cytotoxicity rAAV2-retro virus, have achieved deep analysis of gene profiles with projectome information [21,22]. Yet, the spatial locations of the neurons and the architecture of the network are still lacking, hampering the comprehensive understanding of the organization principles and wiring logic of neural networks.

The prefrontal cortex (PFC) is a critical integration center within the brain network, responsible for a wide range of essential functions including cognition, decision-making, memory and emotions [23,24]. Dysfunctions in PFC circuitry and its related functions can lead to various cognitive and neuropsychiatric disorders [25,26]. Single-cell RNA sequencing and spatial-omics analysis have revealed the sophisticated cellular architecture of the PFC across different species [27–29]. Additionally, the global PFC neural network has been investigated by several technologies such as fMOST and the combination of neural circuit tracing and single-cell sequencing provide valuable insight into the neural connections between the PFC and other brain regions [22,30,31]. However, the multi-modal PFC atlas encompassing neuronal connectivity, transcriptomes, and spatial organization remains fragmented and lacks all-inclusive integration. This hampers the deep analysis of the organizational logic of PFC neural circuits and their biological functions. For instance, how are the diverse neurotransmitter and neuropeptide receptors configured within distinct circuits to specifically decode the neural input signals? What is the organization and synchronization logic of different projectomic and transcriptomic cell clusters, and the molecular mechanism underlying the wiring of these circuits?

The aim of this study is, therefore, to develop a cost-effective, high-throughput neural circuit tracing method with the robust capacity to simultaneously decipher neural projectome, transcriptome and spatial organization information at single-cell resolution. With the combination of barcoded tracing virus, single-cell sequencing and spatial-omics, we developed a robust Single-cell Projectome-transcriptome In situ Deciphering Sequencing (SPIDER-Seq) method. Leveraging SPIDER-Seq, we delineated an atlas of mouse PFC integrating projectomics, transcriptomics and spatial-omics information (33 766 cells for single-cell sequencing and 124 829 cells for spatial-omics). This publicly available multi-modal dataset (https://huggingface.co/spaces/TigerZheng/SPIDER-web) offers an unprecedented view of the neural circuitry in the PFC and sheds deep insight into the neural circuit-specific gene expression pattern, spatial distribution, neural transmission information and neural wiring organizing principles of mouse PFC. Notably, the multi-modal dataset generated by SPIDER-Seq can be trained to predict PFC neuron projections with high accuracy by combining gene profiles and spatial information via machine learning.

## RESULTS

### Deciphering the PFC spatial projectome and transcriptome architecture by SPIDER-Seq

**Figure 1.**
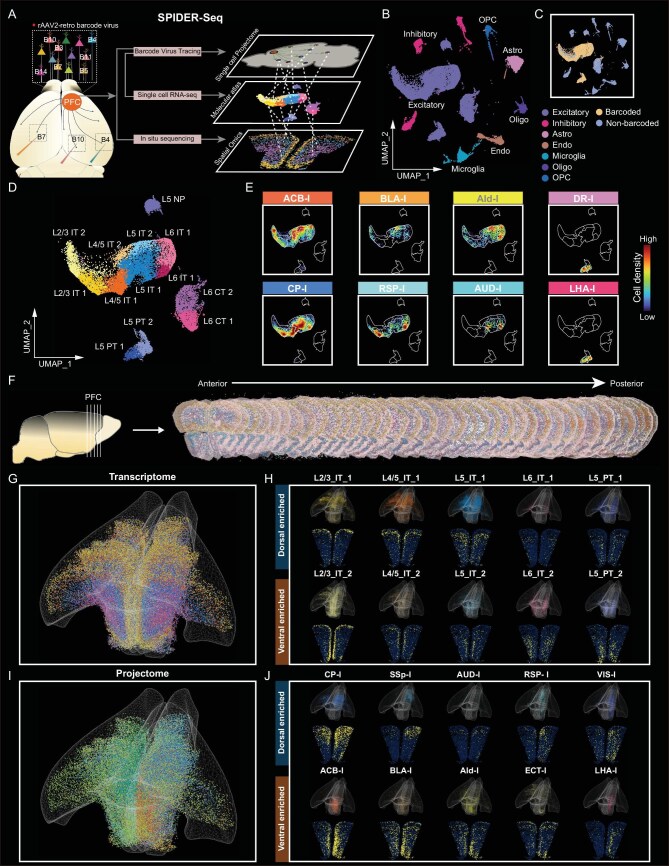
Delineating multi-modal PFC atlas embedding single-cell projectomics, spatial-omics and transcriptomics by SPIDER-Seq. (A) Schematic diagram of the SPIDER-Seq workflow. The rAAV2-retro barcode viruses were injected into different nuclei of the mouse brain to achieve high-throughput single-cell resolution retrograde tracing of the PFC projection circuits. Thirty days post-injection, single-cell RNA sequencing and *in situ* sequencing were performed on the PFC tissue to decipher the projectome with transcriptomic and spatial information. (B) UMAP of all cells in scRNA-seq, colored by main cell clusters. (C) Distribution of barcoded cells in UMAP plot. (D) UMAP plot shows PFC excitatory neurons clustered into 13 unique subtypes. (E) Density scatter visualization on UMAP of neurons projecting to different downstream targets. Points are colored by cell density. (F) *In situ* sequencing of barcodes and marker genes of excitatory neuron subtypes in 36 consecutive coronal slices of PFC (Bregma: 2.8 mm–0.5 mm). (G) 3D visualization of the spatial distribution of PFC excitatory neuron subtypes, which are displayed separately in (H) and Fig. S1K. (H) Spatial distribution of different excitatory neuron subtypes in 3D (top) and 2D (bottom) within an example slice (Bregma: 2.1 mm). (I) 3D visualization of the spatial distribution of neurons projecting to 15 downstream targets, which are displayed separately in (J) and Fig. S1K. (J) Spatial distribution of neurons projecting to different targets in 3D (top) and 2D (bottom) within an example slice (Bregma: 2.1 mm).

Despite the rapid advance of projectome-transcriptome decoding methods, there are still multiple urgent needs for improvement in: (1) reducing the cytotoxicity of tracer viruses which could significantly induce gene profile alterations; (2) the high-resolution detection of spatial location and the surrounding local environment information of the neurons in the network; (3) integrating multi information modalities in the same sample; (4) simultaneous high multiplex target tracing. To help address these requirements, we developed SPIDER-Seq, a cost-effective method to achieve high-throughput, single-cell resolution tracing of projection neurons along with transcriptional and spatial profile in mouse PFC (Fig. 1A). First, we generated the retrograde rAAV2-retro tracing virus library containing diverse DNA barcodes (Fig. S1A), of which the barcode sequences are listed in Table S1. To validate the specificity of the rAAV2-retro-barcode virus, we injected the virus into the ventral striatum (ACB) and examined the cellular distribution patterns using fluorescence *in situ* hybridization. As shown in Fig. S1B, barcode signals were predominately observed in excitatory neurons (barcode merged with *Slc17a7* positive cell), this is consistent with the view that the long-range projection neurons in the cortex are predominantly excitatory neurons [16]. Moreover, we also validated that one cell can be infected by more than 10 different rAAV2-retro viruses *in vitro* (data not shown), which was also confirmed by the following *in vivo* experiments (Fig. S1C).

Next, the unique barcoded viruses were injected into 24 main downstream targets of mouse PFC, covering the majority of the whole projection outputs of the PFC according to the Allen Mouse Brain Connectivity Atlas. After extensive optimization, we achieved precise injections in up to 16 nuclei within the same brain, enabling us to redundantly cover all 24 target nuclei with multiplexed injections in just three mice (Table S2), in which the reproducibility was validated through subsequent SPIDER-Seq analysis. The validation of fluorescence of injection sites is demonstrated in Fig. S2. We waited 30 days post-injection to allow for the complete expression of the barcode, and then performed single-cell sequencing on the retrogradely labeled PFC tissue. To further delineate the spatial architecture of the PFC transctiptome and projectome, we performed *in situ* sequencing for 47 sequences including 32 excitatory neuron subtype marker genes and 15 circuit tracing barcodes across 36 continuous slices spanning the entire PFC (Fig. 1F). As the marker genes are quite unique or predominantly expressed in different excitatory neuron subtypes, the combination of these marker genes and the barcode information could be enough to differentiate the neurons’ subtypes and align these omics data (Fig. S5B). The integrated analysis of multi-modal data from SPIDER-Seq provided an atlas with spatial organization, molecular landscape, anatomy and the projectome information of the projection neurons (33 766 cells for single-cell sequencing and 124 829 cells for spatial-omics) in the PFC at single-cell resolution.

Through single-cell RNA sequencing, 33 766 high-quality cells were clustered into 7 main types (Fig. 1B and S1D–S1F). Among these, 18 615 neurons (4964 genes per neuron) were further classified into 11 types, consistent with a previous study [27] (Fig. S1G). Next, we conducted elbow analysis to carefully filter out background barcode UMIs (Fig. S3A and S3D), and obtained 9038 barcode-labeled cells projecting to 24 downstream targets (Fig. 1C and S3B–S3C). The barcode-labeled cells were predominately excitatory neurons, and its proportion to non-neurons was below 0.5% (Fig. S1H), supporting the integrity of our data and the analysis pipeline. Of note, we observed that 67.2% of the neurons target more than one nucleus (Fig. S1C), with neurons being co-infected by up to 13 barcoded viruses. This indicated that our approach can be effectively applied for multiplex downstream target nuclei tracing.

Next, the excitatory neurons were further clustered into 13 transcriptomic subtypes. Except for NP (near-projecting) subtypes, neurons in each layer were categorized into two distinct subtypes (Fig. 1D and S1I), suggesting the presence of transcriptomic differentiation within each layer of PFC neurons. According to the viral barcode information, we mapped the PFC projection neurons targeting to the 24 downstream nuclei onto this detailed transcriptomic atlas. Our data revealed that the neurons targeting the same downstream nucleus are distributed across multiple transcriptomic subtypes, with the neurons targeting to different downstream nuclei exhibiting distinct distributions in the transcriptomic atlas (Fig. 1E and S1J).

Next, we performed *in situ* sequencing for 47 genes by multiplex detection (Fig. S4) based on the modified MiP-seq protocol [32]. Overall, we obtained 124 829 PFC neurons with spatial information, of which 76 512 were barcode-labeled cells. By Tangram analysis [33], we integrated the single-cell RNA sequencing data with the *in situ* sequencing data, thereby delineating the spatial transcriptomic architecture of the PFC (Fig. 1G and S5A). The spatial transcriptome data revealed a spatial gradient distribution pattern of the excitatory transcriptomic cell clusters (Fig. 1H and S5B–S5C) together with marker genes within each layer of the PFC (Fig. S5D). For example, both IT and PT neuron clusters exhibit a dorsal to ventral subtype separation trend in each layer (Fig. 1H). Based on this spatial transcriptomic information, we categorized PFC IT and PT neurons into dorsal-enriched and ventral-enriched cell clusters, respectively (Fig. 1H). Meanwhile, we constructed a three-dimensional (3D) spatial projectome map of the projection neurons targeting 15 downstream targets in the same mouse with single-cell resolution (Fig. 1I–J and S1K). Notably, the spatial projectome also revealed a similar dorsal-ventral distribution trend, suggesting a potential interlink between transcriptome and projectome in the PFC.

To validate the accuracy of *in situ* sequencing, we first compared the marker gene distribution patterns of our data with the Allen ISH atlas, which showed consistent results (Fig. S5C). Then we compared the distribution of viral barcode signals obtained by *in situ* sequencing with the florescencent rAAV2-retro tracing results in indpendent mice, and repeat measurements in another mouse. The consistent distribution patterns in Fig. S6 demonstrate the accuracy and reproducibility of our barcode detection. Meanwhile, as the barcode signal retrogradely traced from LHA, one of the PT neuron projecting targets, should be located in layer L5, we validated its colocalization with the layer L5 pyramidal neuron specific marker gene *Pou3f1* by FISH assay. As shown in Fig. S7, the barcode signals that retrogradely traced from LHA are indeed mainly merged with *Pou3f1* in layer L5, supporting the specificity integrity of our experiments.

Next, we conducted further systematic quality control, reproducibility and integrity analysis of SPIDER-Seq. First, we compared the differences between barcoded and non-barcoded neurons to evaluate the impact of viral infection on gene expression of the infected neurons. The Pearson correlation between these two groups is 0.98 (Fig. S1L), indicating that the effect of rAAV2-retro virus infection on gene expression is negligible. Importantly, we also compared the projection patterns and intensity between different replicates resolved by SPIDER-Seq, yielding an overall Pearson correlation between different samples of 0.81 (Fig. S1M), which supported the reproducibility of our viral infections and data interpretation. Moreover, we compared the projection profiles resolved by single-cell sequencing and *in situ* sequencing, revealing a Pearson correlation of 0.80 (Fig. S1N), further demonstrating the reproducibility between these two independent approaches. Additionally, we validated our PFC projection profiles resolved by SPIDER-Seq with the fMOST data from Gao *et al*.’s study (Table S6) [31], achieving a Pearson correlation of 0.81 (Fig. S1O). Together, these data demonstrated the reproducibility and integrity of our SPIDER-Seq data. This robust method may greatly facilitate delineating the brain multi-modal network atlas and understanding neural circuit organization logic.

### Spatial and transcriptomic configuration of PFC projectome by SPIDER-Seq

**Figure 2.**
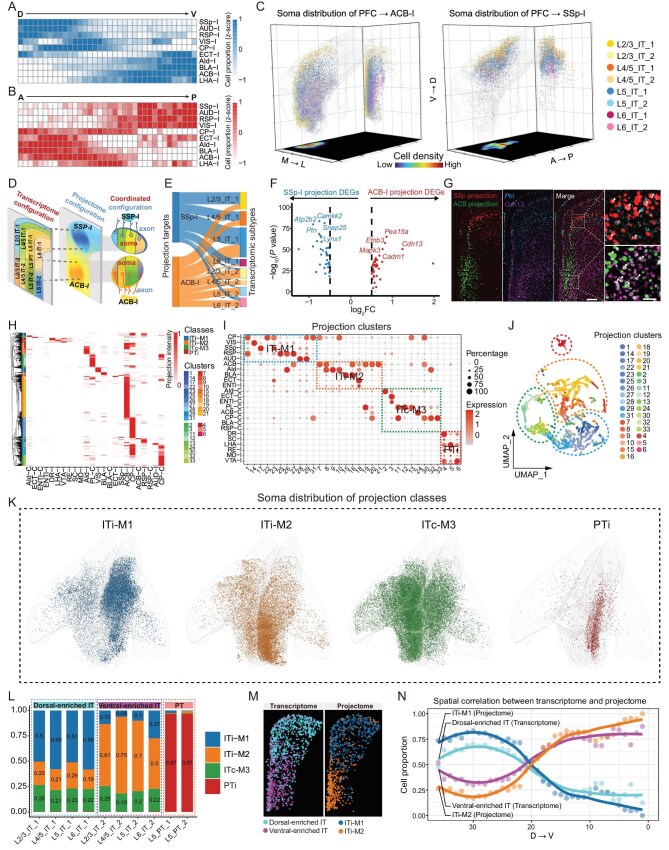
Deciphering the spatial and transcriptomic configuration of the PFC projectome by SPIDER-Seq. (A) Heatmap showing the proportion of PFC neurons projecting to different downstream nuclei from dorsal to ventral (D to V) axis. The cell proportions were normalized by row to calculate the *z*-score. (B) Heatmap showing the proportion of PFC neurons projecting to different downstream nuclei from anterior to posterior (A to P) axis. The cell proportions were normalized by row to calculate the *z*-score. (C) 3D visualization of the spatial distribution of PFC neurons projecting to ipsilateral ACB-I and SSp-I, colored by transcriptomic subtypes. Right grid shows all neurons superimposed on the coronal plane. Bottom grid shows the density of all neurons on the transverse plane. (D) Schematic diagram showing the layered distribution of PFC transcriptome and the spatial gradient organization across layers of projectome. (E) Sankey diagram showing the transcriptomic subtype composition of PFC neurons projecting to ACB-I and SSp-I, respectively. (F) Differentially expressed genes (DEGs) between ACB-I and SSp-I projection neurons. (G) *In situ* hybridization assay showing that the ACB-I enriched gene *Cdh13* accumulated in the ventral PFC, whereas the SSp-I enriched gene *Ptn* accumulated in the dorsal PFC (Bregma: 2.1 mm). Blue, *Ptn*, purple, *Cdh13*, red, SSp-I, green, ACB-I. Scale bar: 500 *µ*m. Magnified view of the white boxed area (right). Scale bar: 50 *µ*m. (H) Heatmap of the projection intensity of 9038 PFC projection neurons to 24 targets. Each row represents the projection intensity of a single neuron. Neurons are divided into four projection classes and 33 projection clusters by hierarchical clustering. (I) Dot plot shows the projection of 33 projection clusters to 24 targets. The color and size represent projection intensity *z*-score and cell percentage, respectively. The dashed box highlights four projection classes. (J) UMAP analysis of the PFC projection matrix showing the distribution of 33 projection clusters. These projection clusters can be divided into four projection classes: PTi, ITi-M1, ITi-M2 and ITc-M3. (K) 3D visualization of the spatial distribution of four projection classes in the PFC. (L) Proportion of transcriptomic subtypes of the four projection classes, colored by projection class. (M) Spatial distribution of dorsal-enriched IT and ventral-enriched IT transcriptomic subtypes (left). Spatial distribution of ITi-M1 and ITi-M2 projection classes (right) (Bregma: 2.1 mm). (N) Spatial correlation between transcriptome and projectome of IT neurons. The color of lines correspond to (M).

The SPIDER-Seq multi-modal PFC atlas embedding large scale neural circuit, transcriptome and spatial architecture information at single-cell resolution provides a unique opportunity to understand the underlying cellular and circuital organization logic. To interpret the relationship among projectome, transcriptome, anatomy and the spatial organization principles, we first analyzed the architecture of the projection neurons. We observed that neurons targeting different downstream nuclei exhibit unique spatial distributions. For example, neurons targeting SSp-I, AUD-I, RSP-I and VIS-I are predominantly located in the posterior dorsal part of the PFC, whereas neurons targeting ECT-I, AId-I, BLA-I and ACB-I are found mainly in the anterior ventral part (Fig. 2A and B). As illustrated in Figs 2C–D and S8A, the projection neurons targeting to ACB-I and SSp-I gradiently occupied different territories in the 3D space of the PFC and consist of different transcriptomic cell subtypes, respectively. These data may illuminate how the projection neurons synchronously configure their soma (for transcriptome) and axon (for projectome) in the PFC. The 3D atlas of the projectome targeting each nucleus together with the transcriptome of each excitatory subtype are shown in Video 1. These data suggested that the spatial transcriptome and projectome were geographically configured within the PFC with distinct organization principles.

With the integrated transcriptome information, SPIDER-Seq can also reveal the transcriptomic differences between various types of projection neurons. For example, the neurons targeting SSp-I and ACB-I not only exhibit different spatial distributions, but also show distinct transcriptomic subtype compositions. Neurons from L2/3IT1, L4/5IT1 and L6IT1 predominantly target SSp-I, while those from L2/3IT2, L4/5IT2, L5IT2 and L6IT2 prefer to target ACB-I (Fig. 2E). Additionally, the SSp-I projecting neurons express significantly high levels of *Snap25, Camkk2* and *Ptn*, while the ACB-I projecting neurons highly express *Pea15a, Efnb3* and *Cdh13*, which was validated by FISH assay (Fig. 2F and G).

We also observed specific gradient spatial distribution patterns of neurons projecting to different regions of striatum. Neurons projecting to the ipsilateral ventral striatum (ACB-I) were primarily located in the ventral region (Fig. 2C and S8C), while those projecting to the ipsilateral dorsal striatum (CP-I) tended to concentrate in the dorsal region (Fig. S8B and S8C), which is consistent with a previous study [34]. Meanwhile, we found transcriptomic differences between the neurons targeting the ventral and dorsal striatum as shown in Fig. S8D. These data highlighted the high throughput capability of SPIDER-Seq to systematically analyze neuronal projection patterns and gene expression as well as spatial organization at the single-cell level.

To further analyze the detailed projection patterns of PFC neurons, we performed hierarchical clustering on the single-cell projection profile of each neuron, resulting in the identification of 33 distinct projection clusters (Fig. 2H–J). These projection cell clusters can be grouped into four projection classes: PTi, ITi-M1, ITi-M2 and ITc-M3, each displaying distinct 3D spatial distribution patterns within the PFC (Fig. 2K and S8E–S8F). PTi were clustered into a separate class, sending projections to subcortical regions such as LHA-I, VTA-I, DR-I, *et al.* IT neurons were further characterized based on their projection patterns as follows: (1) ITi-M1 class mainly project to the ipsilateral dorsal striatum (CP-I) and medial and dorsal cortical regions including RSP-I, VIS-I, AUD-I and SSp-I, of which the somas are located in the dorsal part of the PFC. Anatomically, ITi-M1 was mainly restricted to ACC; (2) ITi-M2 class primarily project to the ipsilateral ventral striatum (ACB-I), BLA-I, and lateral cortical regions including AId-I, ECT-I and ENTI-I, of which the somas are located in the ventral part of the PFC, around PL and IL anatomic regions; (3) ITc-M3 neurons primarily project to the contralateral brain regions including AId-C, ECT-C, ENTI-C, PL-C, ACB-C and CP-C (Fig. S8G).

Next, we performed spatial projectome and transcriptome integrated analysis on these projection classes and clusters, and identified distinct differentially expressed genes in each clusters (Fig. S8H–S8J). Consistent with previous data [17], neurons in the PTi class are pyramidal tract neurons originating from layer 5, largely composed of L5-PT-1/2 transcriptomic clusters (Fig. 2L). In contrast, the IT classes comprise neurons from various transcriptomic clusters across different layers (Fig. 2L). While most projection clusters contain neurons from multiple layers, several projection clusters exhibit strong preferences for their transcriptomic cell type composition. For example, 75.0% of neurons in projection cluster 3, which simultaneously target ECT-C, AId-C and LENTI-C, belong to L2/3-IT transcriptomic clusters. Similarly, 69.2% of neurons in projection cluster 26, targeting RSP-I and AId-I, consist of L6-IT transcriptomic clusters (Fig. S8K and S8L). The identification of the projection specific transcriptomic signature genes would help PFC researchers develop new genetic tools to target and manipulate those cells to better understand the functions of specific circuits.

We also observed transcriptomic differences between ITi-M1 and ITi-M2 classes. ITi-M1 projecting neurons are primarily composed of dorsal-enriched transcriptomic clusters, while ITi-M2 projecting neurons are mainly derived from ventral-enriched transcriptomic clusters (Fig. 2L). Notably, the spatial distribution of ITi-M1 and ITi-M2 projection classes is highly correlated with spatial gradients of the dorsal- and ventral-enriched transcriptomic cell clusters (Fig. 2M and N), suggesting a synchronized configuration of different neuron projection clusters and transcriptomic cell types in the PFC.

### Spatial, anatomic and trascriptomic configuration of PFC IT projection neurons

**Figure 3.**
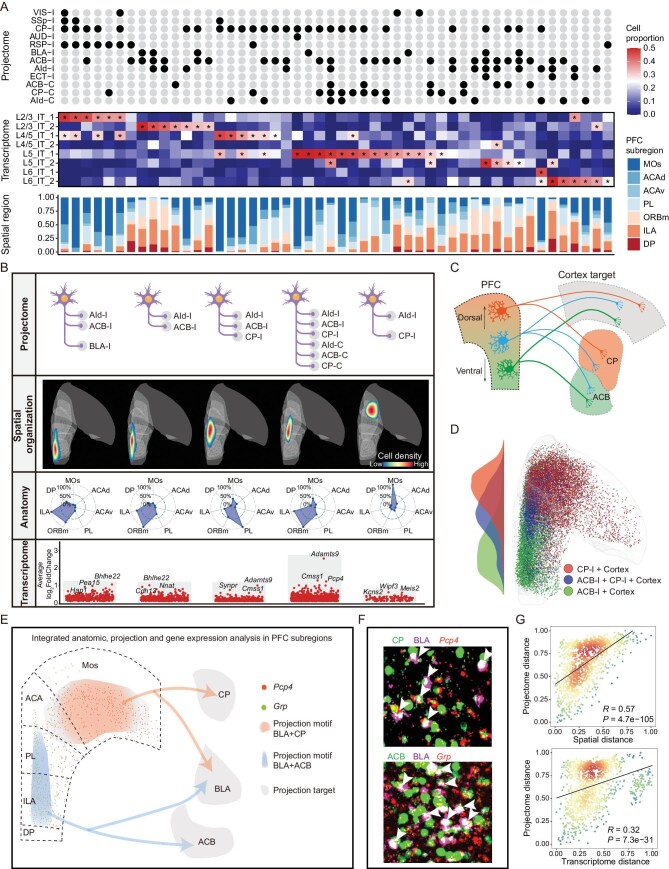
Integrative analysis of spatial and transcriptomic organization pattern of PFC IT projection neurons. (A) Integrative analysis of PFC IT projection motifs, transcriptome subtypes, and spatial distribution. The top 50 projection motifs based on binarized projection matrix (top), transcriptomic subtypes (middle), and spatial distribution in different PFC anatomical subregions (bottom) of individual projection motifs. Asterisks: cell proportion larger than 0.25. (B) Transcriptional, anatomic and spatial information of different PFC IT projection motifs targeting AId-I. Top, projection motifs. Upper middle, spatial distribution. Lower middle, anatomy composition. Bottom, differentially-expressed genes (DEGs) of 5 AId-I projection motifs. (C) Schematic diagram showing the spatial distribution of the neurons co-projecting to cortex (or BLA) and striatum (CP or ACB or both). (D) Spatial distribution of neurons with CP-I + Cortex (or BLA) (red), ACB-I + Cortex (or BLA) (blue), and ACB-I + CP-I + Cortex (or BLA) (green) projection motifs. (E) Integrated anatomic, projection and gene expression analysis in PFC subregions. The distribution of two projection motifs targeting BLA is consistent with DEGs. (F) Barcode neurons of two projection motifs (BLA + CP, BLA + ACB) merged with DEGs. (G) Correlation between projectome and spatial location (top) or transcriptome (bottom). Each point represents the spatial or transcriptome euclidean distance (x-axis) and projection euclidean distance (y-axis) between a pair of projection motifs in (A), colored by scatter density.

Given the high diversity in the projections of IT neurons, we further analyzed the spatial and cellular configuration principles of PFC IT projection neurons. To quantify the diversity of IT projection patterns, we binarized the projection to downstream targets, and presented the landscape of the transcriptomic cell type composition and spatial information of the top 50 IT projection motifs (Fig. 3A). We exemplified five projection motifs targeting AId-I to demonstrate their detailed spatial, transcriptomic and anatomic configuration. Different projection motifs targeting AId-I exhibit a gradient spatial transition from dorsal to ventral regions correlated to the axon branching locations in the striatum (ACB-I or CP-I; Fig. 3B). Neurons projecting to both AId-I and ACB-I are mainly located in the ventral part of the PFC, while those targeting both AId-I and CP-I predominantly distribute in the dorsal part of the PFC, and the triple-targeting neurons (AId-I, CP-I and ACB-I) are located in an intermediate zone, with distinct transcriptomic signatures, respectively (Fig. 3B and S9A). Similar patterns also appear in the BLA-I targeting projection motifs, as shown in Fig. S9B and S9C.

To further test whether this pattern represents a common organization principle, we systematically analyzed the spatial architecture of all the cortex and striatum co-targeting IT neurons in the PFC. In our dataset, 84% of IT neurons that project to the cortex/BLA also extend their axons to the striatum (CP or ACB; Fig. S9D). These neurons exhibit distinct spatial distributions and spatial transcriptomic signatures depending on their targeting in the striatum. Projection neurons co-targeting the dorsal striatum (CP) are mainly distributed in the dorsal part of the PFC, while those co-targeting the ventral striatum (ACB) are primarily enriched in the ventral part. Projection neurons that simultaneously target both ACB and CP are predominantly located in the intermediate transitional zones (Fig. 3C and D). Correspondingly, these projection neurons showed distinct gene profiles, of which some transcriptomic signatures also display a dorsal-enriched to ventral-enriched gradient, as confirmed by FISH assay (Fig. S9E–S9G).

We also conducted an integrated anatomy, projection and transcriptomic analysis and identified that CP and BLA projection neurons are mainly located in the Mos region of the PFC and highly expressing *Pcp4*, while the ACB and BLA projection neurons are mainly located in PL and ILA regions in the PFC highly expressing *Grp*, which was further confirmed by FISH assay (Fig. 3E and F). Importantly, our analysis revealed significant correlations between neuron projections and their gene profiles, and spatial location, respectively (Fig. 3G). Together, these data suggest that although the projectomic and transcriptomic cell clusters display distinct spatial organization principles, they are synchronously configured in the PFC. Our analysis could provide unprecedented integrated anatomic and transcriptomic formation at projection motif levels, which may greatly contribute to understanding the function of each anatomic region of the PFC.

### Co-projection principle of PFC IT neurons

**Figure 4.**
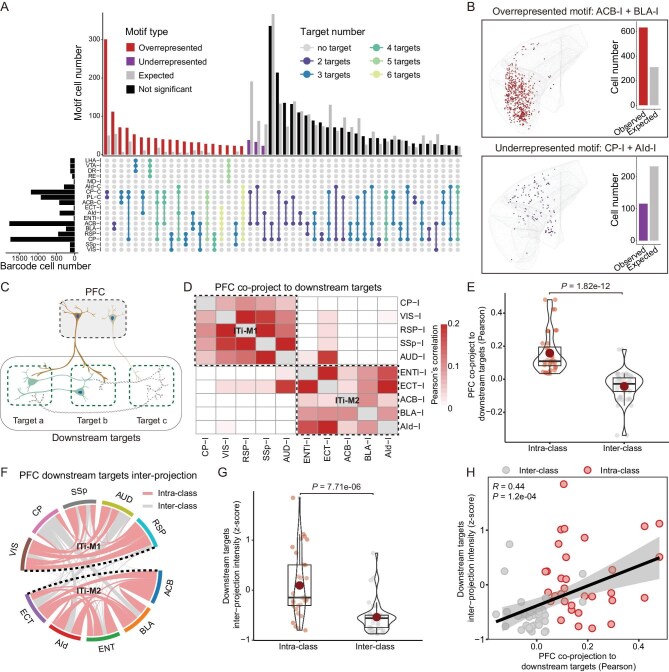
Wiring logic analysis of PFC IT co-projection neurons. (A) Upset plot showing the observed and expected cell numbers for each projection motif. We compared the observed cell numbers of projection motifs with the expected null model to calculate significance. Red, purple and black bars represent overrepresented, underrepresented and not significant, respectively. Gray bars represent expected. Different projection motifs are colored according to the target number. (B) Example of spatial distribution of overrepresented (ACB-I + BLA-I) and underrepresented (CP-I + AId-I) motifs. The barplot showing the observed and expected cell numbers for motifs in the spatial dataset. (C) Schematic diagram showing that the downstream targets co-projected by PFC IT neuron share more circuit connections. (D) Heatmap showing the co-projection probability of downstream targets targeted by PFC IT neuron. Each tile represents the Pearson correlation of two targets co-projected by PFC projection neurons. (E) Box and violin plot showing that the co-projection probability within projection classes is significantly higher than that across classes. (F) Circuit connection intensity between downstream targets targeted by PFC IT neurons; connectivity intensity refers to the density of AAV-labeled nerve from the Allen Mouse Brain Connectivity Atlas. The red arcs represent intra-class projection, and the grey arcs represent inter-class projection. (G) Box and violin plot showing that circuit connection intensity between downstream targets within the same projection classes is higher than that across classes. (H) Correlation between co-projection probability and circuit connection intensity between downstream targets targeted by PFC IT neurons.

Our data indicated that the majority of barcoded neurons target two or more nuclei simultaneously (Fig. S1C). To examine whether these downstream targets are randomly associated together or sophisticatedly organized with certain kinds of logic, we aligned the projection patterns of multiple targeting neurons resolved by SPIDER-Seq with the expectations of random association (Fig. 4A and S10A). The results suggested that these multiple-projections are not randomly configured. For example, the multiple-projection neurons targeting both ACB-I and BLA-I are overrepresented, whereas the multiple-projection neurons targeting CP-I and AId-I are underrepresented (Fig. 4B and S10B).

Furthermore, we investigated the wiring principle of multi-projection neurons in the PFC. First, we analyzed co-projection probability to the downstream targets from different PFC projection neurons. These results showed that downstream targets belonging to the same projection class (ITi-M1 or ITi-M2) have significantly higher probability to be co-projected compared to targets from different classes (Fig. 4D and E). Considering that high co-projection probability may accompany the engagement of more simultaneous information processing and subsequent information exchange, the co-projected downstream targets should presumably have more reciprocal circuit connections (Fig. 4C). Thus, we collected the projection data for the corresponding nuclei from the Allen Mouse Brain Connectivity Atlas and calculated the intensity of the connectivity between downstream targets (Table S7). The results verified that the downstream targets from the same projection class do indeed have significantly more intensive circuit connections (Fig. 4F–G and S10C–S10D). Additionally, there is a positive correlation between the connectivity intensity of downstream nuclei and the probability of co-projecting by PFC neurons (Fig. 4H). These findings revealed one wiring principle in the PFC and validated that projection of the PFC neurons are indeed sophisticatedly organized rather than randomly wired together.

### Configuration of neural signal decoding and transmission machineries in the content of PFC neural network

PFC neurons decode upstream information inputs through neurotransmitter and neuropeptide receptors, transmitting signals to downstream networks by releasing neurotransmitters and neuropeptides. Deciphering the dynamic and sophisticated neural signal transmission flow in neural networks is the prerequisite for the understanding of neural computation and brain functions [35,36]. However, the detailed signal decoding and transmission processes remain elusive. Our SPIDER-Seq dataset contains a high quality of gene profile data which reach to 4964 genes per neuron and have important expression information including low-expression genes such as the receptor genes for neurotransmitters and neuropeptides. This empowered us to systematically delineate the expression patterns of the various neural signaling related molecule genes in the content of the PFC network, which could bring unprecedented insights into the logic of neural signal encoding in different circuits.

**Figure 5.**
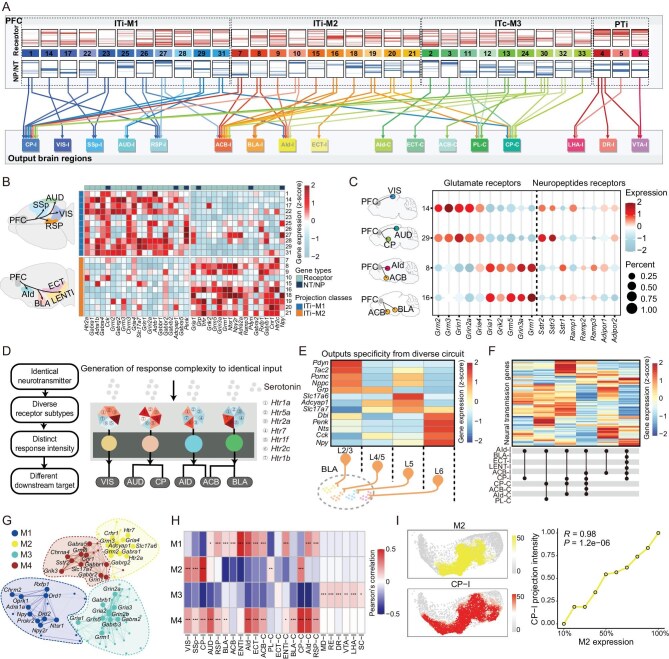
Configuration of neural signal decoding and transmission machineries in the content of the PFC neural network. (A) Neural signal decoding machineries and transmission flow in PFC projection clusters. The upper panel showing 33 projection clusters grouped by four projection classes. Heatmaps showing the expression of the genes encoding neurotransmitters (NTs) (blue), neuropeptides (NPss) (blue) and their receptors (red) in each projection cluster. The lower panel shows the downstream targets. Arrows represent the output of each PFC projection cluster to different downstream targets, colored by projection clusters. (B) The heatmap displays the differential expression of neural signaling molecules in two projection classes of IT neurons targeting the ipsilateral brain regions (ITi-M1 and ITi-M2). (C) Different projection clusters expressing diverse neurotransmitter (glutamate) and neuropeptide (somatostatin, SST; adrenomedullin, ADM; adiponectin, ADIPOQ) receptor subtypes. (D) Schematic diagram showing that PFC projection clusters decode serotonin signals by different expression levels and combinations of receptor subtypes. (E) Neurotransmitter transporter and neuropeptide precursor gene expression in different layers of neurons targeting the BLA-I. (F) Different projection motifs targeting the AId-I have different neural signaling molecule expression patterns. (G) Co-expression network of genes related to neural signaling molecules. Each node represents a single gene, and edges represent co-expression links between genes. Genes are divided into four co-expression modules. The top 10 hub genes per module are labeled. (H) Heatmap showing the correlation between projection and different gene co-expression modules. Each tile in the heatmap represents the Pearson correlation coefficient between the projection intensity of a target and the expression of a co-expression module. Statistical significance was determined using two-sided Fisher’s exact test, *P < 0.05, **P < 0.01, ***P < 0.001. (I) An example showing that gene co-expression module M2 and projection to CP-I share similar distributions on transcriptome UMAP (left); correlation between M2 expression and CP-I projection intensity (right).

Hence, we depicted the neural signaling molecule heatmaps for different projection clusters based on the expression of neurotransmitter transporters, neuropeptide precursors and their receptor genes (Fig. 5A and S11A). The heatmaps revealed significant differences in the expression of neural signaling molecules between PT and IT projection neurons, including glutamate receptors (*Gria4, Grm3*), serotonin receptors (*Htr2a, Htr5a, Htr1b*), dopamine receptors (*Drd1*), various neuropeptide receptors (*Mc4r, Mchr1, Npr3, Cckbr*), as well as neuropeptide precursors (*Pdyn, Adcyap1, Grp, Npy, Penk, Cck*) and neurotransmitter transporter (*Slc17a6*) genes (Fig. S11B). These differences may underly the distinct functions of these two types of PFC neurons. Next, we analyzed the neural signaling molecular expression pattern of two IT projection classes targeting the ipsilateral brain areas. Our data showed that the neurons projecting to the dorsal cortical regions/dorsal striatum and those projecting to the lateral cortical regions/ventral striatum showed distinct gene expression patterns of neural signaling molecules (Fig. 5B). To this end, previous studies have demonstrated the functional separation of PFC projections to the dorsal medial and the ventrolateral cortex as well as the dorsal and ventral striatum [24,34].

As the different expression levels and combinations of neurotransmitter receptor subtypes may reflect the differential decoding of the same neurotransmitter input [37,38], we then examined the expression patterns of neurotransmitter receptor subtypes in different projection clusters. As shown in Fig. 5C, glutamate receptor subtypes are distinctly expressed across different projection clusters. The dorsal cortical regions targeting projection clusters, such as cluster 14 (mainly targeting VIS) and 29 (mainly targeting AUD and CP), showed significantly higher expression levels of *Grm2, Grm3, Grin1, Grin2a* and *Gria4*. In contrast, projection cluster 8 (mainly targeting AId and ACB) and projection cluster 16 (mainly targeting BLA and ACB), highly express *Gria1, Grik2, Grm5, Gria3a* and *Grm1*. This phenomenon has also been observed in other neuromodulatory receptors, such as neuropeptide receptors (Fig. 5C) and serotonin receptors (Fig. S11C).

The neighborhood neurons distributed in the same spatial territory in the PFC may likely receive the same local inputs, but could project to different downstream targets. Thus, it would be important to distinctly encode the same inputs into more diverse signals via the combination of differentially expressed signal decoding receptor genes in different projection motifs. By this principle, an identical input has the capacity to encode diverse kinds of downstream signals in the neural network, which may contribute to the generation of enough signal complexity to match the sophisticated information transmission capacity of the brain (Fig. 5D).

The release of neurotransmitters and neuropeptides is the primary means by which neurons transmit signals [39]. We observed that the mRNA expression levels of different neurotransmitter transporter and neuropeptide precursor genes vary across different projection clusters (Fig. 5B). Notably, projection neurons in distinct layers in the PFC targeting the same nucleus show significant differences in the expression of neurotransmitter transporters and neuropeptide related genes (Fig. 5E). For example, IT projection neurons targeting BLA from L2/3 highly express *Pdyn* and *Tac2*, neurons from L5 highly express *Adcyap1, Slc17a7* and *Slc17a6*, whereas neurons from L6 highly express *Npy* and *Penk*. The molecular configuration of this kind of expression pattern may allow the same downstream target nucleus to unambiguously differentiate inputs from different upstream nuclei, which might be important to guarantee the specificity of the neurotransmission in the intricate neural network.

Furthermore, we analyzed the expression of neural signaling molecules including neurotransmitter transporters, neuropeptide precursors and related receptor genes in six projection motifs targeting AId-I and observed significant differences in their expression patterns in each projection motif (Fig. 5F). Similar patterns also appeared in the RSP-I targeting projection motifs, as shown in Fig. S11D. These data suggest that diverse PFC projection circuits are equipped with differential neural signal decoding and transmission machineries, thereby facilitating their functional diversity. We then investigated whether there are co-expression patterns of the neural transmission molecules in different PFC neural projection clusters. By co-expression analysis, we identified four co-expression modules of the neural signaling related molecules (Fig. 5G and S11E–S11G). Importantly, our data revealed a high correlation between the projection patterns and the co-expression of neural signaling molecules (Fig. 5H). For example, the projection intensity of neurons targeting CP-I is positively correlated with the expression intensity of co-expression module M2 (Fig. 5I). These results suggested that the expression pattern of diverse neural signaling molecules in different PFC circuits is not randomly organized; rather, it forms sophisticated and specific co-expression modules in different circuits, underlying the synergistic action among different neural signaling molecules.

### Correlation of neural circuit wiring molecules with projection patterns

**Figure 6.**
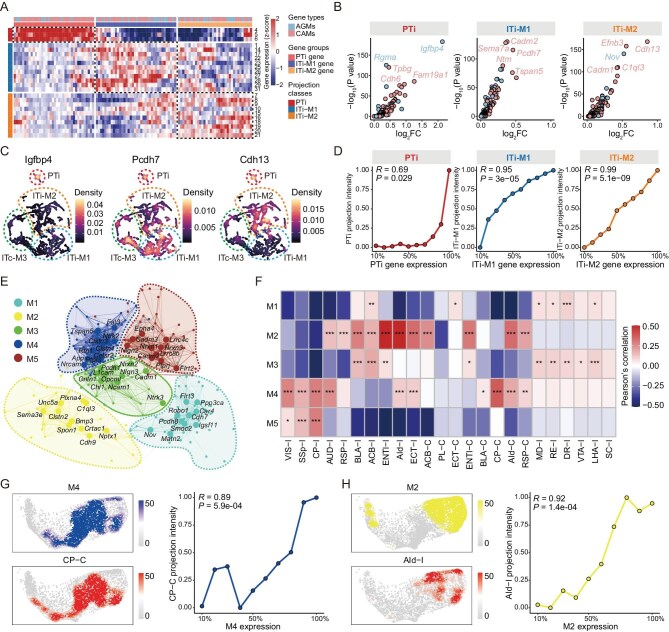
Correlation between expression of neural circuit wiring molecules and PFC projection patterns. (A) Expression of molecules related to neuronal circuit wiring molecules (AGMs: axon guidance molecules, CAMs: cadherin molecules) in different projection clusters. The dotted boxes outline the gene groups associated with projection classes. (B) Volcano plot showing genes related to neural circuit wiring enriched in PTi, ITi-M1 and ITi-M2 projection classes, respectively. (C) Projection UMAP shows the expression distribution of three genes related to neuronal circuit wiring (*Igfbp4* enriched in PTi, *Pcdh7* enriched in ITi-M1 and *Cdh13* enriched in ITi-M2). (D) Correlations between gene expression of three gene groups and PTi, ITi-M1 and ITi-M2 projection classes, respectively. (E) Co-expression network of genes related to circuit wiring. Each node represents a single gene, and edges represent co-expression links between genes. Genes are divided into five co-expression modules. The top 10 hub genes per module are labeled. (F) Heatmap showing the correlation between projection and different gene co-expression modules. Each tile in the heatmap represents the Pearson correlation coefficient between the projection intensity of a target and the module eigengenes (MEs) of a co-expression module. Statistical significance was determined using two-sided Fisher’s exact test, *P < 0.05, **P < 0.01, ***P < 0.001. (G) An example showing that gene co-expression module M4 and projection to CP-C share similar distributions on transcriptome UMAP (left); the correlation between M4 expression and CP-C projection intensity (right). (H) An example showing that gene co-expression modules M2 and projection to AId-I share similar distributions on transcriptome UMAP (left); the correlation between M2 expression and AId-I projection intensity (right).

Although the expression of wiring-related genes during development is crucial for neural circuit formation, the expression of specific genes (such as cadherins and axon guidance molecules) in adulthood is critically important for circuit maintenance. To explore the relationship between projection patterns and these molecules, we further analyzed the high-quality transcriptome profiles of PFC projection neurons in the content of neural circuits. Our data revealed that different projection classes exhibited distinct expression patterns of neural circuit wiring molecules (Fig. 6A and S12A). For example, *Igfbp4, Rgma* and *Fam19a1* are highly expressed in PT neurons (Fig. 6B and C). In addition to the significant differences between PT and IT projection neurons, projection neurons within different IT classes, such as ITi-M1 and ITi-M2, also showed differential gene expression (Fig. 6A). For instance, *Cadm2, Sema7a* and *Pcdh7* are highly expressed in the ITi-M1 class, whereas *Efnb3, Cdh13* and *Nov* are highly expressed in the ITi-M2 class (Fig. 6B and C). Importantly, the expression of these wiring molecules is in proportion to the intensity of the projection classes (Fig. 6D).

Next, we analyzed whether the projection patterns of PFC neurons are interrelated to the co-expression of the neural circuit wiring molecules. We first analyzed the co-expression patterns of neural circuit wiring genes across all barcoded neurons, yielding five distinct co-expression modules (Fig. 6E and S12B–S12D). The relationship between projection patterns and gene co-expression modules was further analyzed, which revealed numerous significant correlations between the projection patterns and the strength of gene co-expression in specific modules (Fig. 6F). For example, the CP-C projection is highly related to the co-expression of the M4 module, of which the projection intensity shows a positive correlation with the expression strength of M4 module genes (Fig. 6G). Similarly, the projection intensity targeting AId-I is significantly related to the expression strength of M2 module genes (Fig. 6H). Together, these results suggested that the neuronal projection patterns are closely related to the co-expression of molecules associated with cadherins and axon guidance.

### Predicting PFC neuron projection patterns using integrated transcriptomic and spatial information

The distinct expression patterns of neural circuit formation and maintenance related genes, such as cadherins and axon guidance genes in different projection classes, inspired us to predict the projection pattern by gene profile via machine learning. While the gene profile can predict the projection pattern to a certain degree [22,29], the accuracy needs to be signicantly improved. Our data showed that on top of the correlation between neuron projection patterns with its gene profiles, the projection patterns are also highly related to its spatial location in the PFC (Fig. 3G). Thus, we attempted to train a machine learning model to predict the projection patterns of PFC neurons based on transcriptome and spatial location. First, we integrated the single-cell transcriptomic dataset and spatial-omics dataset from SPIDER-Seq. This enabled us to obtain an integrated transcriptomic dataset with projection and spatial information at the single-cell level. Then, we encoded binary labels (0 for no-projection or 1 for projection) for each targeted nucleus. Finally, we used the transcriptome principal components (PCs) and spatial information (spatial coordinates: X, Y, Z) as input features, and constructed an XGBoost machine learning model to predict the projection information (Fig. 7A).

**Figure:**
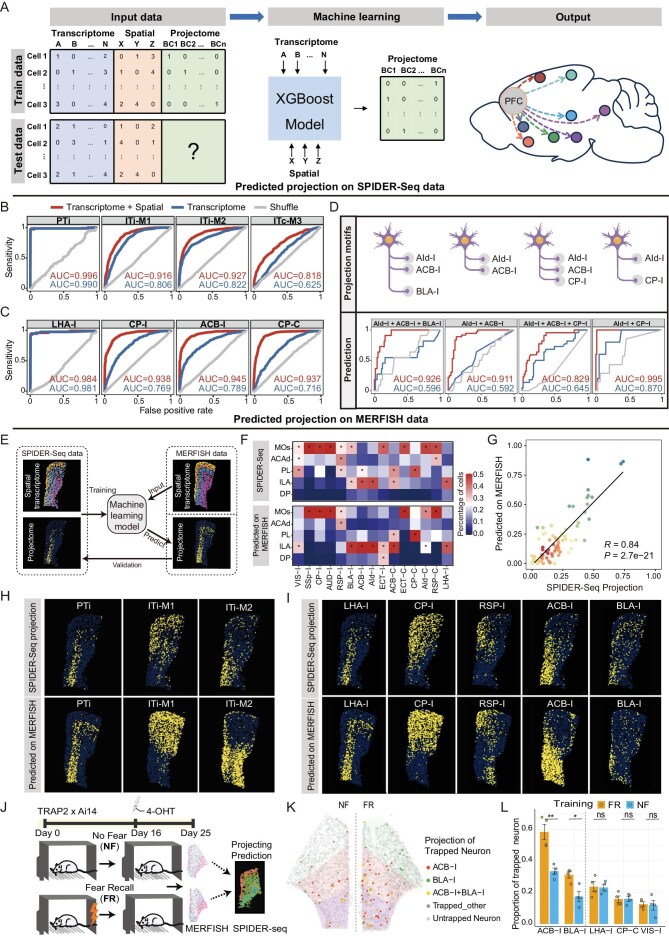
Prediction of neuron projection by integrated gene profiles and spatial location information by machine learning. (A) Schematic diagram of the steps in machine learning modeling. The training data (including transcriptome, spatial location and projectome) was used to train an XGBoost model. The test data (including only transcriptome and spatial location) was used as the input of the machine learning model and projectome information as the output. (B) ROC curves for four predicted projection classes. The red curves unitize both transcriptomic and spatial profiles as input, the blue curves unitize only transcriptomic profile as input, and the gray curves are random shuffle control. (C) ROC curves for four predicted projection targets. The meaning of the curve color is the same as (B). (D) ROC curves for four predicted AId-I projection motifs. The meaning of the curve color is the same as (B). (E) Schematic diagram of predicted projection on MERFISH data. Our SPIDER-Seq data (including transcriptome, spatial location and projectome) was used to train an XGBoost model. MERFISH data (including only transcriptome and spatial location) was used as the input of the machine learning model and projectome information as the output. Validation was performed by comparing the projectome predicted on MERFISH with the projectome mapped by SPIDER-Seq. (F) The percentage of neurons projecting to different nuclei in PFC subregions mapped by SPIDER-Seq (top); the percentage of neurons projecting to the putative projecting nuclei predicted by machine learning model based on the data from the corresponding MERFISH slice (bottom). Asterisks: percentage >0.25. (G) Correlation between the predicted projections based on MERFISH data and the projections revealed by SPIDER-Seq. Each point represents the percentage of neurons in a PFC subregion for a target in (F). (H) The spatial distribution of neuron soma in different projection classes mapped by SPIDER-Seq (top), and predicted by the machine learning model based on MERFISH data (bottom) (Bregma: 1.78 mm). (I) The spatial distribution of neuron soma projecting to different targets mapped by SPIDER-Seq (top), and predicted by the machine learning model based on MERFISH data (bottom) (Bregma: 1.78 mm). (J) Re-illustration of experimental design from Sun *et al*. TRAP2 × Ai14 mice were subjected to either a fear recall (FR) or no-fear (NF) conditioning model; 4-hydroxytamoxifen (4-OHT) was administered on Day 16 to label behavior activated neurons. On Day 25, MERFISH spatial transcriptomics was performed on the PFC. The MERFISH dataset were subjected to our machine learning model for projection prediction. (K) Representative MERFISH samples from NF (NF2_r0) and FR (FR2_r1) groups showing behavior activated (trapped) neurons. Predicted projection identities are color-coded: ACB-I (red), BLA-I (green), ACB-I + BLA-I (yellow) (Bregma:1.78 mm). (L) Quantification of the proportion of trapped neurons within each major projection target across four biological replicates. FR group shows significantly higher projections to ACB-I and BLA-I compared to NF (mean ± s.e.m.; *P < 0.05, **P < 0.01; two-sided *t*-test). Other projection targets show no significant differences (ns).

For model evaluation, we split our data into a training dataset (70%) and a test dataset (30%). The model was trained to predict both the overall projection classes (Fig. 7B) and specific projection targets for individual neurons (Fig. 7C and S13A), respectively. The receiver operating characteristic (ROC) curve demonstrated that our model achieved prediction performance with high accuracy. For example, the prediction accuracy of the LHA-I projection pattern reached 98.4%, and ACB-I with 94.5% accuracy. Notably, integrating spatial coordinates as input features significantly improved the model’s performance compared to using transcriptome features only (Fig. 7B and C and S13A). The lower accuracy of ITc-M3 may be due to its relatively less obvious transcriptomic and spatial features, while PTi shows significantly different transcriptomic and spatial features compared to IT neurons, which attributes to its higher prediction accuracy. To further demonstrate the accuracy of our model, we performed predictions at the projection motif level. Notably, our model can accurately predict projection motifs which are indistinguishable at the transcriptome level after integrating spatial information, such as the different projection motifs of AId-I (Fig. 7D).

To further validate the generalization capacity of our model, we tested it on the PFC MERFISH spatial transcriptome dataset from Bhattacherjee *et al.* [29]. First, we searched the MERFISH slice most closely matching our PFC slice (Fig. S13B) and integrated both datasets based on the spatial and transcriptomic information (Fig. S13C and S13D). This allowed us to apply our projection model to the MERFISH data (Fig. 7E). The projection prediction on the MERFISH slices were highly consistent with the real projection experimental data revealed by SPIDER-Seq in the corresponding slice (Fig. 7F). The spatial visualization of the predicted results on MERFISH slices also shows consistent patterns with our SPIDER-Seq experimental data in the corresponding slice (Fig. 7H and I and S13E). Quantification analysis showed that the Pearson correlation between the overall predicted projection on MERFISH slices and the projection patterns on our slice resolved by SPIDER-Seq reached 0.84 (Fig. 7G).

Having verified the accuracy and generalization capability of our model in neural projection prediction, we next applied it to predict the projections of specific neurons under certain behavioral conditions, as a proof of concept. To achieve this, we obtained the MERFISH dataset from a prior study that captured fear recall (FR) engram neurons using the TRAP system. The experimental design of this study is illustrated in Fig. 7J. We aligned these MERFISH slices with our SPIDER-Seq data and integrated spatial and transcriptomic information to predict neuronal projections. Notably, we found that FR engram neurons projected significantly more to the ACB-I and BLA-I regions compared to non-fear (NF) control neurons. In contrast, no significant differences were observed in projections to other targets, such as the LHA, CP and VIS (Fig. 7K and L). This finding aligns with previous reports implicating the invovement of PFC→ACB-I and PFC→BLA-I pathways in fear recall-related behaviors [40–42].

## DISCUSSION

### Deciphering the multi-modal brain atlas by SPIDER-Seq

The neural circuit is an elaborating multi-modal network embedding diverse information such as connectivity, gene profiles, electrophysiology and spatial location. Recent methods, such as BARseq, BARseq2, VECTORseq, Retro-Seq, Epi-Retro-Seq, MERGE-Seq and Projection-seq have revolutionized high-throughput analysis of projectome information with some gene profiles [13,17–19,22]. However, there are still some urgent requirements for improvement in: (1) reducing the cytotoxicity of tracer viruses which induce gene profile alterations; (2) detecting spatial location and the surrounding local environment information of the neurons in the network; (3) integrating multi-modal information; (4) simultaneous high multiplex target tracing. To this end, we developed SPIDER-Seq by combining circuit tracing viral barcoding strategies with single-cell sequencing and spatial-omics, which allows us to integrate the transcriptome, projectome and spatial profiles of the neurons projecting to multiple nuclei with high throughput capacity and single-cell resolution. Integrity of the SPIDER-Seq data was validated by the comparison between our data with previous fMOST [31] and spatial transcriptome data [29], as well as the data from different verifications [34]. This robust and cost-effective method can greatly facilitate delineating the brain multi-modal network atlas and understanding of neural circuit organization logic.

### The cellular and circuital spatial configuration and organization logic of the PFC

Although the molecular, cellular architecture and neural network of the PFC have been extensively investigated, the integrated multi-modal PFC atlas embedding large scale neural circuit, transcriptome and spatial architecture information at single-cell resolution is still lacking, greatly hindering the understanding of the underlying cellular and circuital organization logic. By virtue of SPIDER-Seq, we delineated the detailed molecular, cellular and circuital spatial configuration of the PFC with single cell resolution. Consistent with previous studies, we showed that different transcriptomic cell clusters are arranged in different cortical layers with a certain dorsal-ventral gradient, due to the inside-out development of these cell clusters in each layer [29,43]. In contrast, the architectures of different projection motifs are configured with a distinct geographical logic, in which each projection motif gradiently occupies a specific territory in the 3D space of the PFC. Neurons with the same projection pattern are allocated together in a specific territory and consist of different transcriptomic cell clusters. By this organization principle, a single projection motif can more dynamically and distinctly decode neural signal inputs and produce more kinds of output signals by diverse expression of neurotransmitters/peptides and their receptors in their diverse transcriptomic cell clusters.

Although it has been demonstrated that majority of PFC IT projection neurons showed multiplex targeting and are sophisticatedly organized, the underlying organization logic remains elusive. Our SPIDER-Seq analysis revealed that the downstream nuclei targeted by the same projection class have significant higher probability to be co-projected by the PFC neurons, which may explain the overrepresented projection patterns which have also been observed by several studies [12,44]. Moreover, the downstream nuclei targeted by the same PFC projection class have significantly more intensive reciprocal circuit connections. One possible underlying mechanism might be that the reciprocal connected neurons are more inclined to be both activated, and then simultaneously release certain kinds of axon guidance cues. This might lead to the high probability to induce axon wiring from the same upstream nucleus during development. Functionally, co-projection to the reciprocal connected neurons wiring principle may facilitate information processing and the subsequent information exchange between these simultaneous signal recipients. It would be of great importance to examine whether this is a general logic for the whole sophisticate brain network.

### Specificity and complexity of neural signal decoding and transmission in the content of the PFC neural network

Deciphering the dynamic and sophisticated neural signal transmission flow in the neural network is the prerequisite for the understanding of neural computation and brain functions [35,36]. However, detailed signal decoding and transmission processes remain elusive. Owing to the high-quality single-cell transcriptome dataset integrated with projection information provided by SPIDER-Seq, we can detect low expression genes such as receptor genes in the content of the neural network, which bring unprecedented insights into the logic of neural signal encoding in different circuits. In addition to the distinct neural signaling molecule expression patterns between PT and IT neurons, we also found significant differences in two projection classes of IT neurons, which may reflect the functional separation of PFC projections to the dorsal medial and the ventrolateral cortex as well as the dorsal and ventral striatum [24,34]. Notably, we showed that the expression pattern of diverse neural signaling molecules in different PFC circuits is not randomly organized; rather, it forms sophisticated and specific co-expression modules in different circuits, underlying the synergistic action among different neural signaling molecules.

The PFC expresses a variety of neurotransmitter receptor subtypes to decode the upstream neurotransmitter signal. The different expression levels and combinations of receptor subtypes in PFC projection circuits may reflect the differential decoding of the same neurotransmitter input [37,38]. In this way, we showed that individual PFC projection clusters selectively overexpress different receptor subtypes of the same neurotransmitter, underlying distinct neural signal decoding capacities and functions in different projection clusters. Moreover, for the projection neurons within the same projection motif, our SPIDER-Seq analysis revealed that they are gradiently distributed in the same spatial territory in the PFC and may likely receive the same local inputs. Thus, it would be important to distinctly encode the same inputs into more diverse signals via the combination of differentially expressed signal decoding receptor genes. By this principle, an identical input has the capacity to encode diverse kinds of downstream signals in the neural network, which may contribute to the generation of enough signal complexity to match the sophisticated information transmission capacity of the brain.

The release of neurotransmitters and neuropeptides is the primary means by which neurons transmit signals [39]. We found that different upstream projection neurons targeting the same nucleus showed distinct expression patterns of neurotransmitter-/neuropeptide-related genes, as illustrated by the distinct expression of neural neuropeptide genes in the IT projecting neurons targeting BLA from different upstream locations in the PFC. The molecular configuration of this kind of expression pattern may allow the same downstream target nucleus to unambiguously differentiate inputs from different upstream nuclei, which may be important to guarantee specificity of the neurotransmission in the intricate neural network. Together, our data depicted the landscape of neural information flow of PFC projection neurons and the underlying neural signal transmission molecules in this PFC network. Moreover, we discovered potential neural signal decoding and transmission principles to synchronize the specificity and complexity of neural transmission by sophisticated expression patterns of signal transmission molecules in different PFC projection neurons.

### Predicting PFC neuron projection patterns using integrated transcriptomic and spatial information

Our data revealed that different projection classes in the PFC distinctly express neural circuit formation and maintenance related genes, such as cadherins and axon guidance genes, which inspired us to predict the projection pattern by gene profile via machine learning. Consistent with previous studies [22,29], while the gene profile can predict the projection pattern to a certain degree, the accuracy needs to be further improved. As SPIDER-Seq can also decipher the spatial information of the projection neurons, which is also correlated to the projection pattern, we then integrated the spatial and ample gene profile information at single-cell resolution for machine learning. By this approach, we achieved significantly high accuracy and, importantly, demonstrated the generalization capacity of our model on the MERFISH dataset. Considering that projection neurons in other brain regions should, in principle, also have intrinsic correlations between the spatial gene profiling and projection pattern, our model can be likely used as an important basic foundation for fine-tuning prediction projection patterns in any brain regions using a smaller amount of dataset. Thus, our data may further contribute to investigations such as, predicting neuron projection by spatial gene profiles after Ca2+ imaging or electrorheology experiments, which can integrate function analysis with circuit information.

## LIMITATIONS

Despite the advances of SPIDER-Seq, there are several issues that need to be further improved. (1) Viral labelling coverage: this is limited by the diffusion area in the injection sites, especially for targeting large nuclei such as the CP. To tackle this issue, we performed injections at different depths to increase the coverage area of the virus (see Methods); (2) Viral infection efficacy: our previous work reported that rAAV2-retro has high efficiency for most cortical area labeling, but relatively lower for other regions [45]. To increase the labelling efficacy, we waited for 30 days to reach high infection efficacy. In future, it would be important to engineer a new generation of highly efficient tracing virus; (3) Local circuit mapping: SIPDER-Seq applied for local circuit mapping, such as inhibitory neuron circuits, needs to be subjected to future study; (4) It should be noted that our study used only females, but were compared with male datasets [29,31]. Although, there are no reports indicating differences in PFC circuit connectivity between female and male mice, this will remains an important consideration for future research on sex-related study.

With rapid advancing research in interdisciplinary fields, such as viral bioengineering, *in situ* sequencing methods, imaging technologies, and machine automation, SIPDER-Seq can be further upgraded to delineate the integrated spatial transcriptome and projectome atlas with high throughput, high 3D resolution and more accuratecy multi-modal information. Moreover, the integrated multi-modal neural network will also shed insights into the organization logic and the computation principle of the neural network and ultimately contribute to brain-inspired artificial intelligence.

## MATERIALS AND METHODS

Detailed materials and methods are available in the Supplementary data.

## Supplementary Material

nwag004_Supplemental_Files

## Data Availability

The raw single-cell RNA-seq data are available from GEO (GSE273066). The raw spatial-omics data for this study are available via Hugging Face at https://huggingface.co/TigerZheng/SPIDER-STdata. The processed data ready for exploration can be accessed and downloaded via our interactive browser at https://huggingface.co/spaces/TigerZheng/SPIDER-web (Fig. S14).

## CODE AVAILABILITY

All data were analyzed with standard programs and packages. The codes are freely accessible from: https://github.com/ZhengTiger/SPIDER-Seq.
