# Supplementary material for: Organization of mouse prefrontal cortex subnetwork revealed by spatial single-cell multi-omic analysis of SPIDER-Seq
Source: Natl Sci Rev. 2026 Jan 16;13(5):nwag004. doi: 10.1093/nsr/nwag004 (PMC12988354; doi:10.1093/nsr/nwag004)
Supplement: nwag004_Supplemental_Files [file nwag004_supplemental_files.zip › Supplementary_figures_and_legend_with_Changes_labeled.docx]

**Supplementary figures**


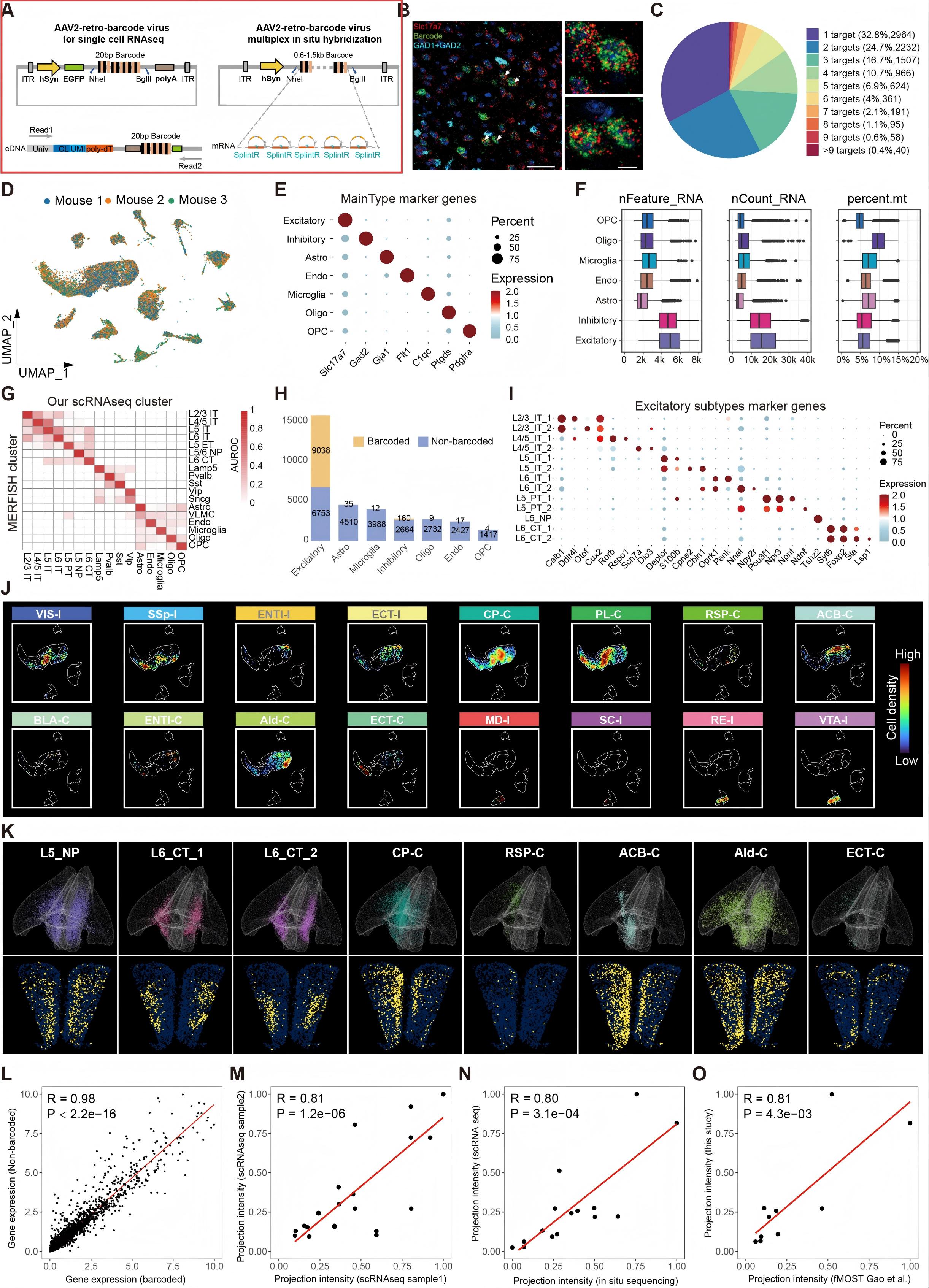


## Figure S1. Delineating multi-modal PFC atlas embedding single-cell projectomics, spatial-omics and transcriptomics by SPIDER-Seq

**(A)** rAAV2-retro-barcode virus designed for single cell RNAseq and schematic of designed primers to recover cell barcode and UMI in read 1, and 3’ tail of EGFP and virus barcode in read 2 (left). rAAV2-retro-barcode virus designed for multiplex *in situ* hybridization and schematic of designed hybridization primers to detect barcode (right).

**(B)** Fluorescence *in situ* hybridization detection shows that the retrograde tracing barcode was mainly distributed in excitatory neurons*.* Excitatory neuron marker: *Slc17a7*, red; Inhibitory neuron markers: Gad1 and Gad2, blue; Barcode signal retrograde tracing from ACB: green. Scale bars, 50 µm. The inset on the right shows a magnified view of the overlap of *Slc17a7* and ACB barcode. Scale bars, 10 µm.

**(C)** Distribution of number of projection targets of PFC barcoded neurons.

**(D)** Integrated UMAP of cells from 3 mouse brains (mouse1:12 targets, mouse2: 14 targets, mouse3: 16 targets (Table S2)), colored by samples.

**(E)** Dotplot showing the expression patterns of maintype marker genes in transcriptome maintypes.

**(F)** Boxplots showing the distribution of the number of genes (left), number of UMI (middle) and mitochondrial genes percentage (right) detected in each transcriptome maintype.

**(G)** Heatmap showing the gene expression correlation between the PFC clusters defined by scRNA-seq in SPIDER-Seq and Bhattacherjee et al..

**(H)** Barplot showing the number of barcoded (yellow) cells in each transcriptome maintype.

**(I)** Dotplot showing the expression patterns of excitatory marker genes in excitatory subtypes.

**(J)** Density scatter visualization on UMAP of PFC neurons projecting to different nuclei.

**(K)** Spatial distribution of different transcriptome subtypes and projection neurons to different nuclei in 3D (top) and a 2D example slice (bottom) (Bregma: 2.1mm).

**(L)** Correlation of transcriptome in barcoded and non-barcoded neurons.

**(M)** Correlation of projectome in different scRNAseq samples.

**(N)** Correlation of projectome in scRNA-seq and spatial-omics data.

**(O)** Correlation of projectome revealed by our SPIDER-Seq and fMOST data.


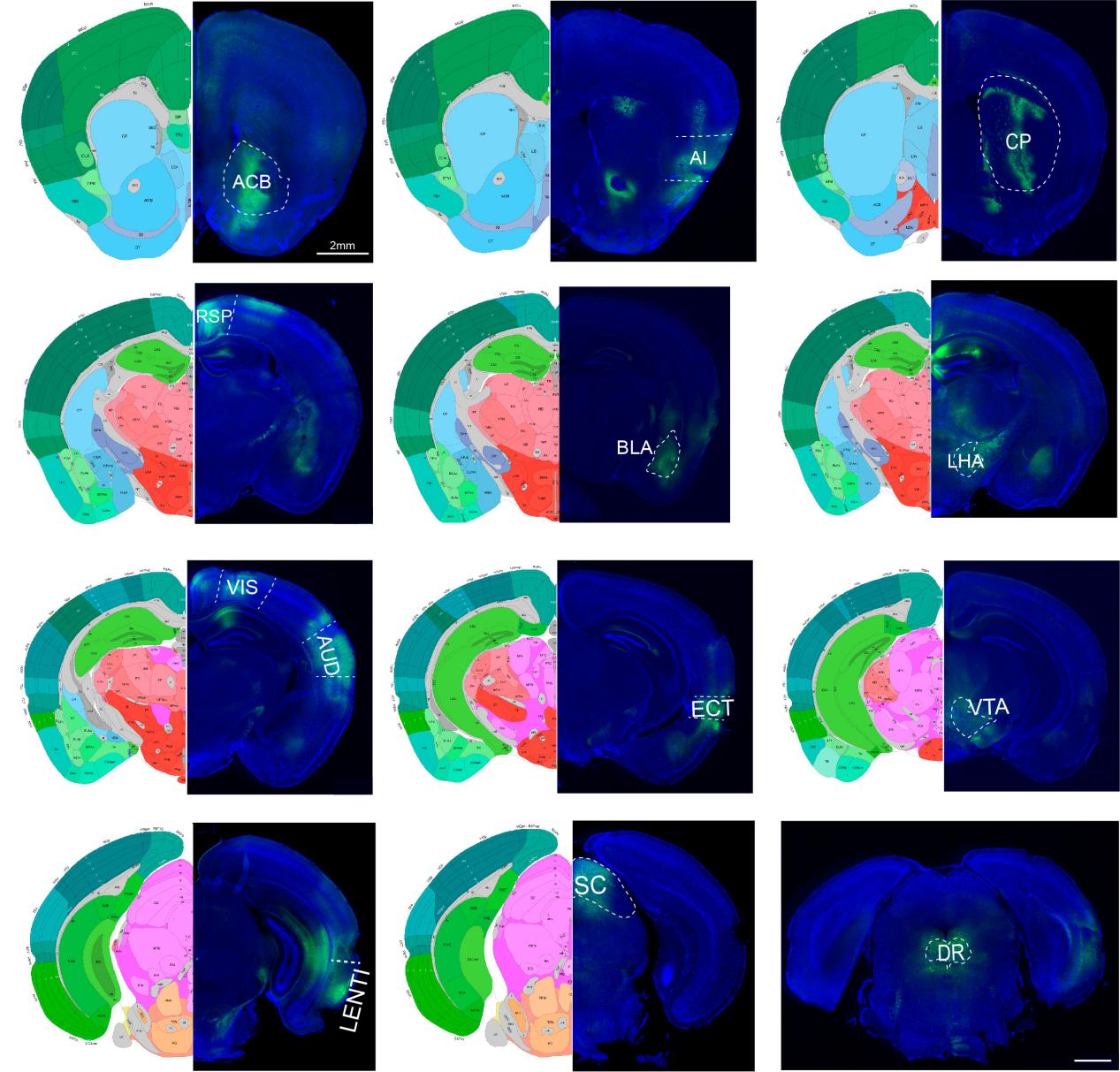


## Figure S2. Injection sites of different nuclei targeted by PFC

Coronal images of the injection sites (ACB, AI, CP, RSP, MD, BLA, LHA, VIS, AUD, ECT, VTA, LENTl, SC, DR) of rAAV2-retro-barcode virus (right) and the corresponding Allen brain atlas (left). Scale bar: 2 mm.


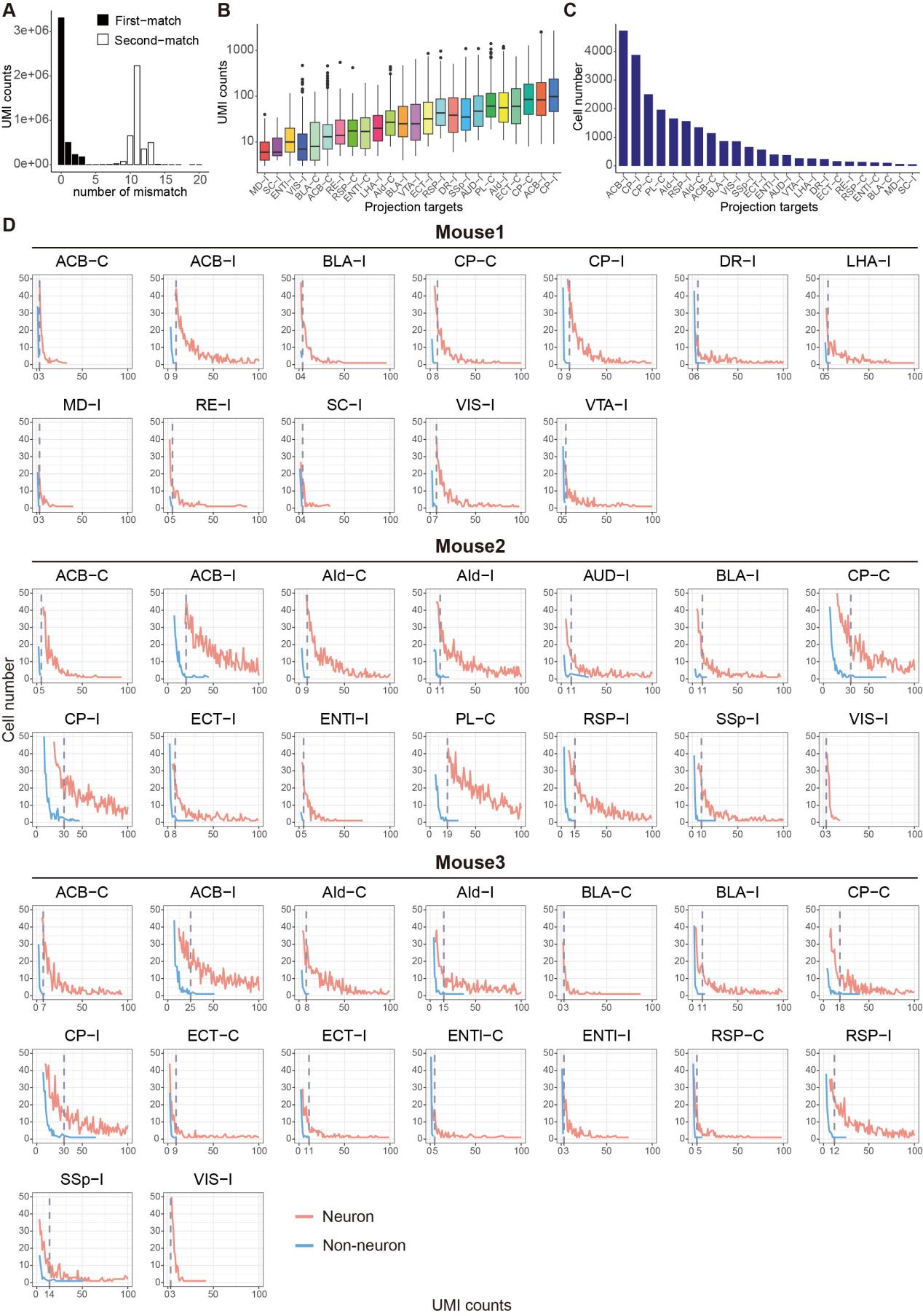


## Figure S3. Extracting projection information from scRNAseq

**(A)** Histogram of the number of mismatches between the First-match reads and the Second-match reads. This indicates that there is enough sequence diversity to distinguish the correct barcodes.

**(B)** Box plot shows the UMI counts for the barcode projecting to different nuclei.

**(C)** Histogram shows the number of barcoded cells for 24 targeted nuclei.

**(D)** The UMI counts and cell number curves for each barcode in 3 scRNAseq samples, grouped by Neuron and Non-neuron. The gray dashed line indicates the threshold of UMI counts for a barcode filter after elbow analysis.


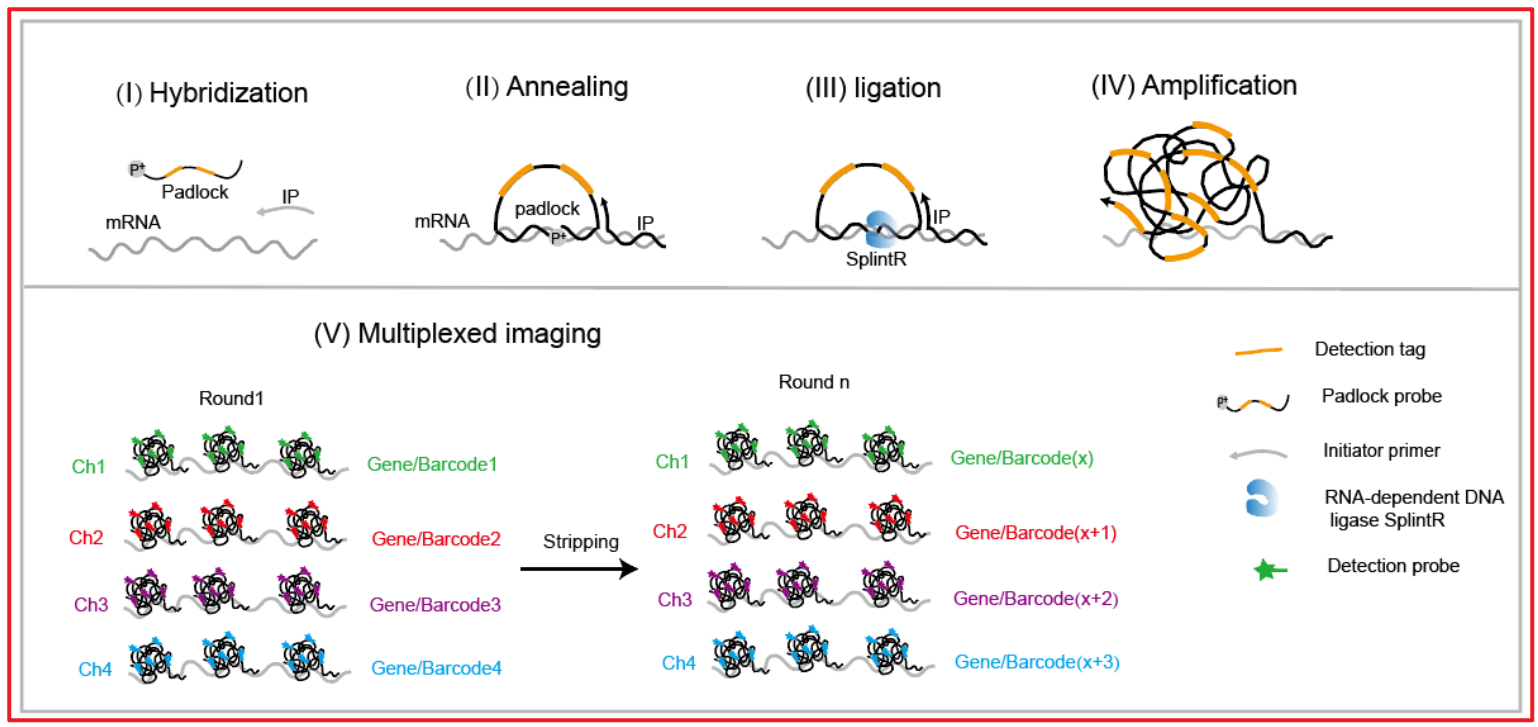


## Figure S4. Flow diagram of multiplex detection based on a modified version of MiP-seq

I. The padlock probe and initiator primer (IP) are hybridized to the target RNA. Notably, the dual barcode primer in MiP-seq is replaced by two repeating detection tag. II. Padlock probe and IP are annealed to the target mRNA to form a padlock structure. III. Padlock probe is circularized by RNA-dependent DNA ligase SplintR to form the RCA template. IV. RCA of circled padlock probe forms rolling-circle products. V. The detection tag is hybridized to the detection probe labelled with fluorescence. After each imaging cycle, probes are stripped away from the tissue using 60% formamide for the next cycle. Five channels for DAPI, 488, cy3, cy5, cy7 are used in each round and each channel represents a detected gene. We detected 47 genes in 12 rounds (**Table S4)**.


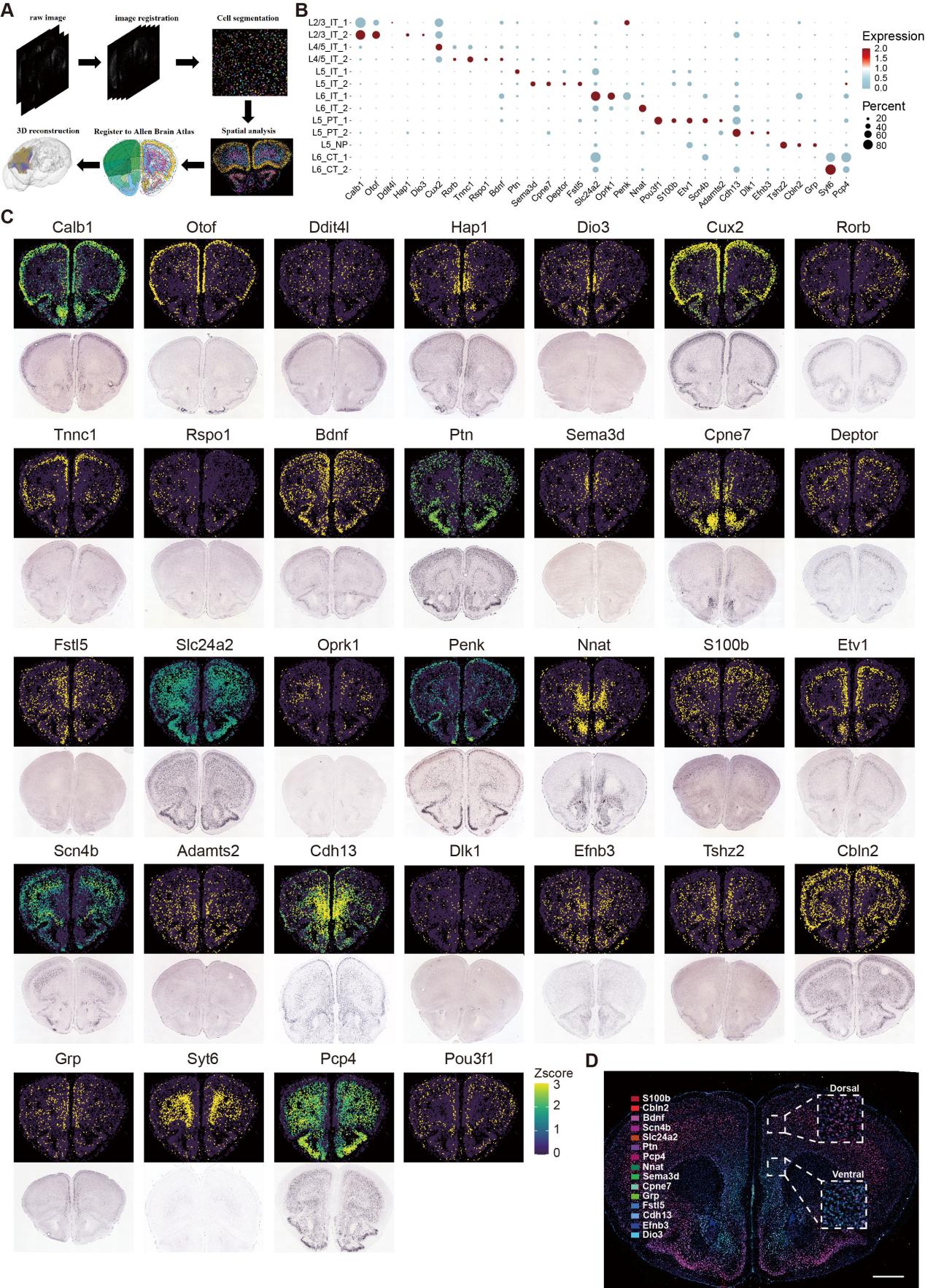


## Figure S5. Spatial gene profiles data analysis of SPIDER-Seq

**(A)** Spatial expression data analysis process of SPIDER-Seq, including image registration, cell segmentation, spatial analysis, registration to Allen brain atlas, and 3D reconstruction.

**(B)** Dotplot showing the expression patterns in excitatory neurons subtypes of 32 marker genes measured by SPIDER-Seq.

**(C)** *In situ* hybridization images show that the expression pattern of 32 markers detected by SPIDER-Seq (top) are consistent with ISH data from Allen Brain Atlas (bottom) (Bregma:2.1mm).

**(D)** The spatial transcription shows spatial gradient separation of the PFC transcription atlas with dorsal-enriched genes (*S100b*, *Cbln2*, *bdnf*, *Scn4*b, *Slc24a2*, *Ptn*, *Pcp4*) and ventral-enriched genes (*Nnat*, *Sema3d*, *Cpne7*, *Grp*, *Fstl5*, *Cdh13*, *Efnb3*, *Dio3*).


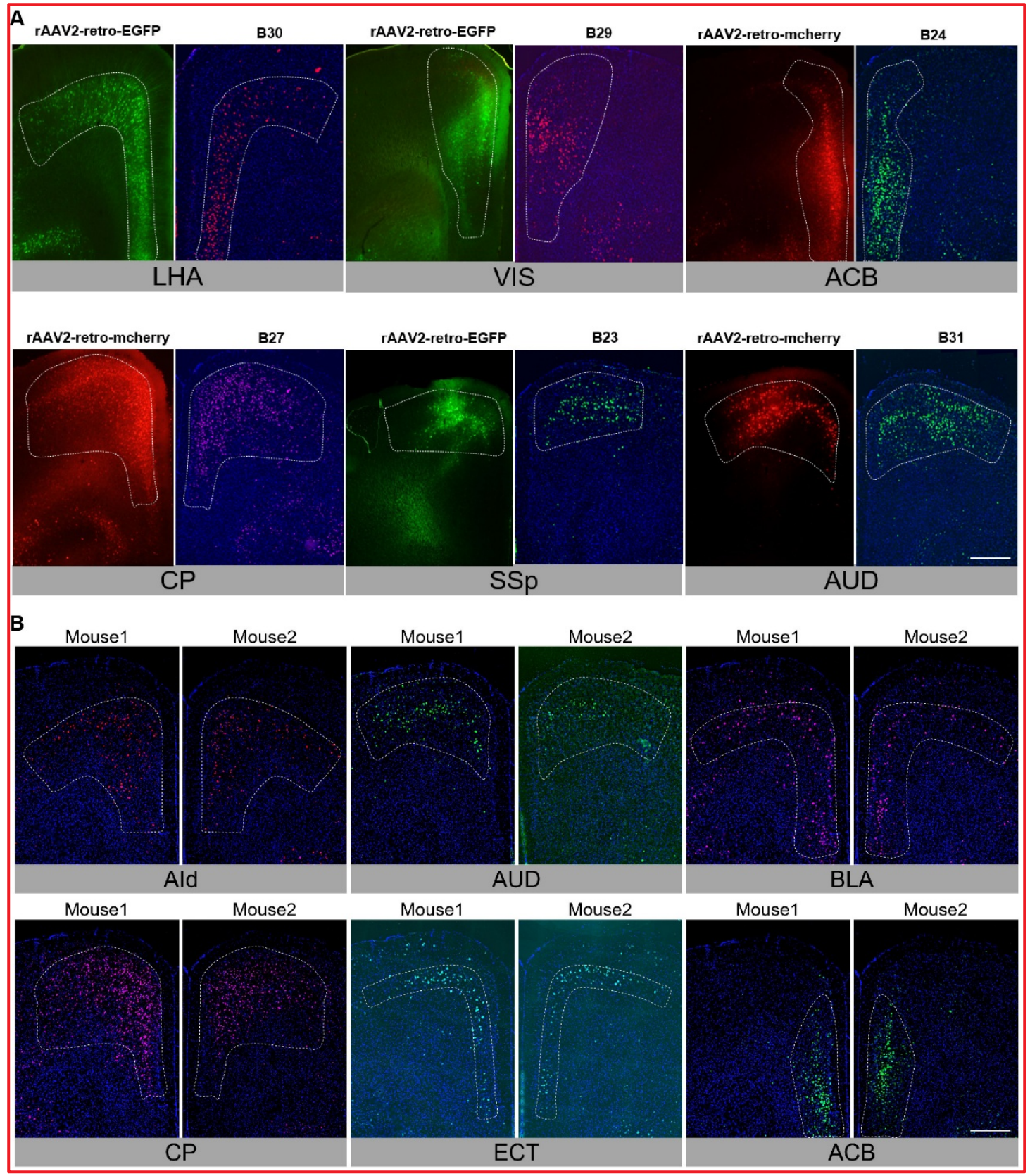


## Figure S6. Comparison the barcode signal distribution obtained by in situ sequencing with fluorescent rAAV2-retro tracing and measurements in another mouse

**(A)** rAAV2-retro expressing mcherry or EGFP were injected into the target region (LHA, VIS, ACB, CP, SSp, AUD) and compare the distribution of fluorescent protein (right) with In situ sequencing signal of barcode (left). Scale bar: 2 mm.

**(B)** Repeat measurements on six target regions (AId, AUD, BLA, CP, ECT, ACB) in another mouse using in-situ sequencing via SPIDER-seq. Scale bar: 2 mm.


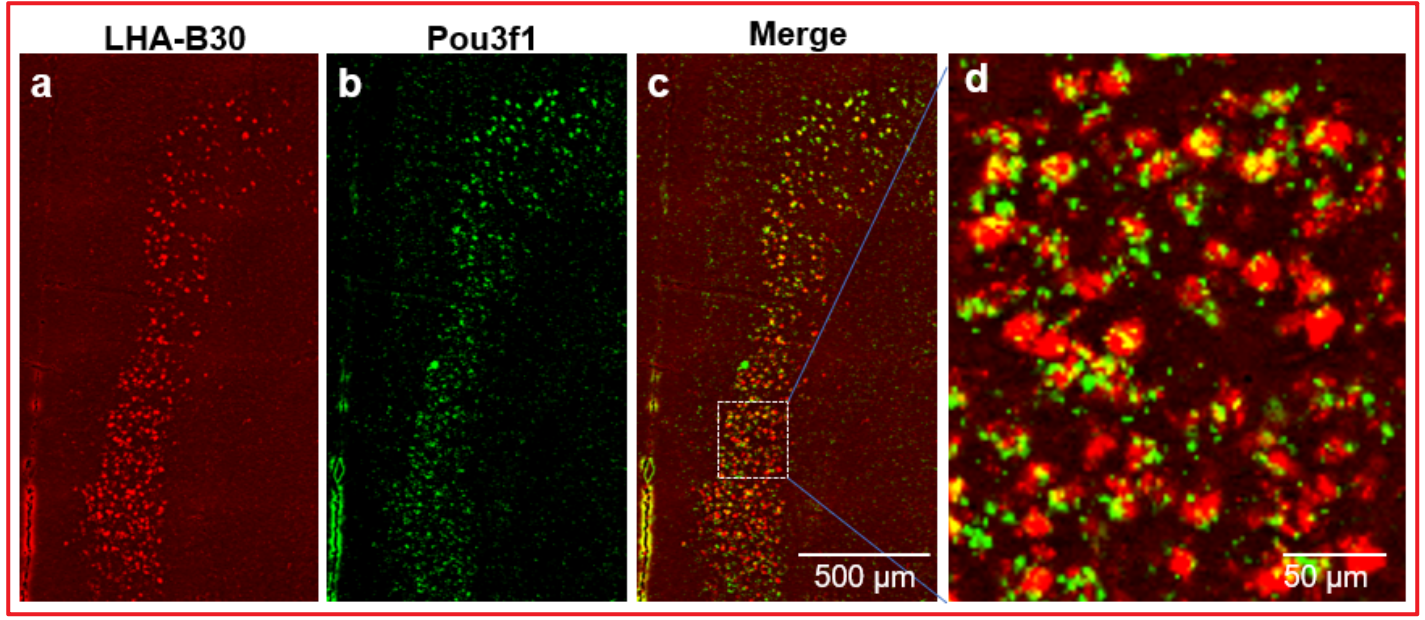


## Figure S7. Barcode that retrogradely tracing from LHA is merged with the pyramidal tract neuron marker gene (Pou3f1)

**(A)** In situ sequencing signal of LHA barcode.

**(B)** In situ sequencing signal of L5 PT Marker gene: *Pou3f1*.

**(C)** Merge of LHA barcode and *Pou3f1*. Scale bar: 500 µm.

**(D)** Magnified view of the white boxed area in c. Scale bar: 50 µm.


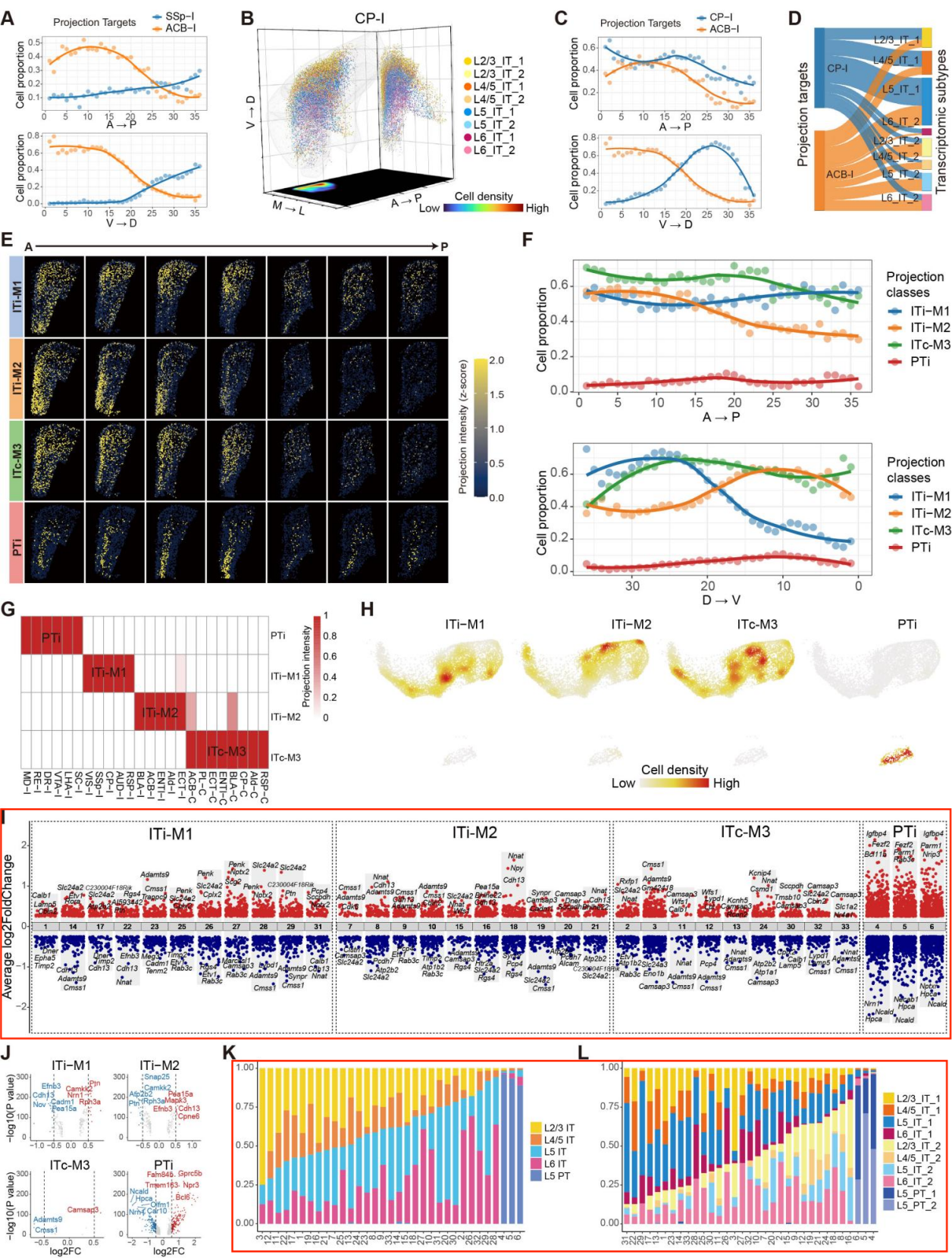


## Figure S8. Deciphering the spatial and transcriptomic configuration of PFC projectome by SPIDER-Seq

**(A)** Distribution curves of PFC neurons projecting to ipsilateral ACB-I and SSp-I along the anterior-posterior axis (top) and the ventral-dorsal axis (bottom), respectively.

**(B)** 3D visualization of the spatial distribution of PFC neurons projecting to ipsilateral CP-I, colored by transcriptomic subtypes. Right grid shows all neurons superimposed on the coronal plane. Bottom grid shows the density of all neurons on the transverse plane.

**(C)** Distribution curves of PFC neurons projecting to ipsilateral CP-I and ACB-I along the anterior-posterior axis (top) and the ventral-dorsal axis (bottom), respectively.

**(D)** Sankey diagram shows the transcriptomic subtypes composition of PFC neurons projecting to CP-I and ACB-I, respectively.

**(E)** Spatial distribution of four projection classes along the anterior-posterior axis.

**(F)** Spatial distribution curves of four projection classes along the anterior-posterior axis (top) and the ventral-dorsal axis (bottom).

**(G)** Heatmap of the projection intensity of four projection classes to 24 targets.

**(H)** Density scatter visualization on UMAP of four projection classes. The color scale represents the density of projection neurons.

**(I)** Differentially expressed genes (DEGs) of 33 projection clusters.

**(J)** Volcano plot showing DEGs in four PFC projection classes.

**(K)** Transcriptome composition of 33 PFC projection clusters, grouped by transcriptomic cell layers.

**(L)** Transcriptome composition of 33 PFC projection clusters, grouped by transcriptomic cell subtypes.

**
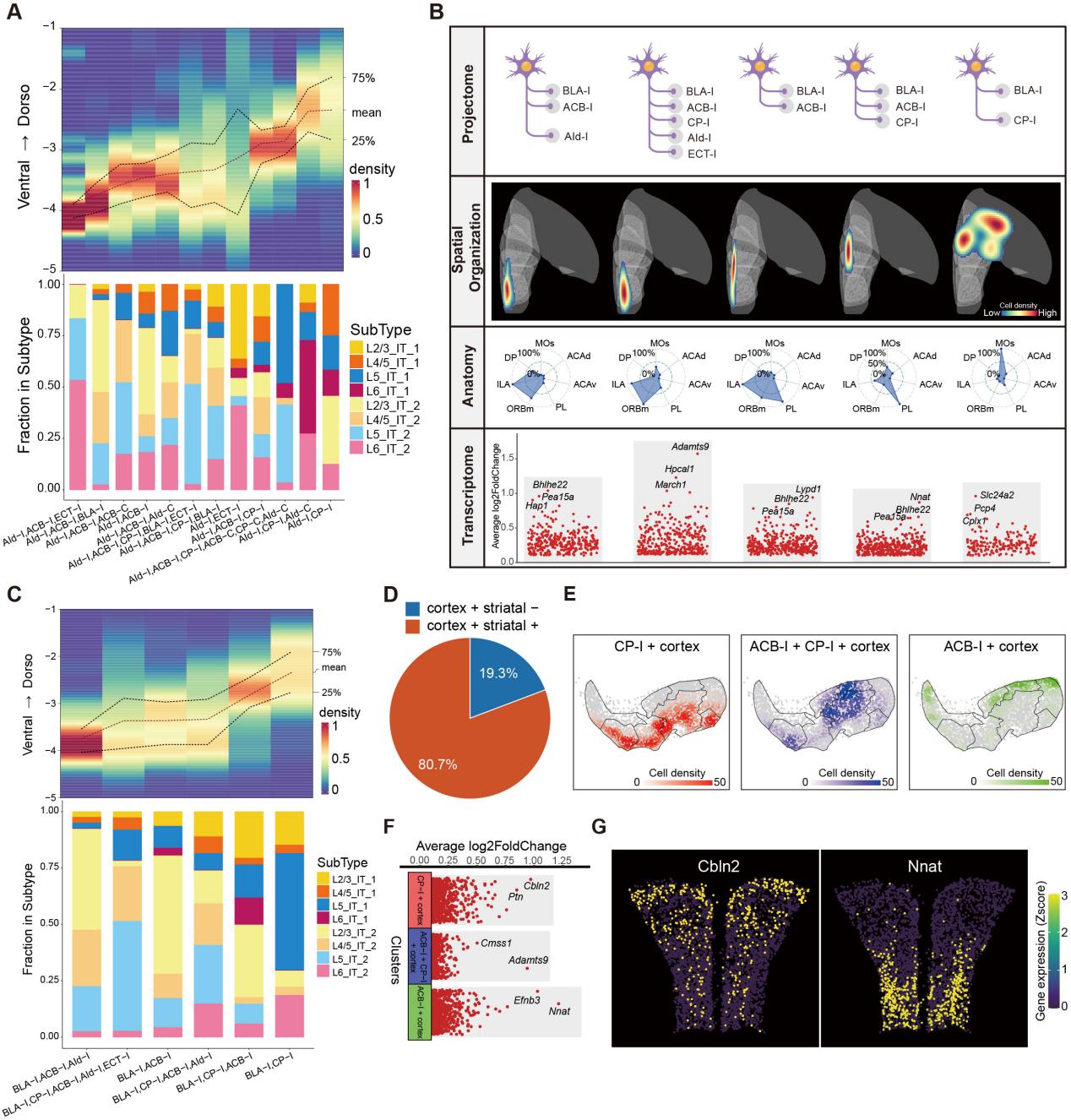
**

## Figure S9. Organization pattern of PFC IT projection neurons

**(A)** Spatial distribution of neurons in different motif targeting AId-I along the ventral-dorsal axis (top), and transcriptome composition (bottom).

**(B)** transcriptomics and spatial information of different PFC projection motifs projecting to ipsilateral BLA. Top, target nuclei. Middle, differentially expressed genes. Bottom, spatial distribution.

**(C)** Spatial distribution of neurons in different motifs targeting BLA-I along the ventral-dorsal axis axis (top), and transcriptome composition (bottom).

**(D)** The percentage of cortex+ striatal- and cortex+ striatal+ projection neurons.

**(E)** Transcriptomic UMAP distribution of CP-I+Cortex (or BLA) (left), ACB-I+Cortex (or BLA) (middle), and ACB-I+CP-I+Cortex (or BLA) (right) projection motifs.

**(F)** DEGs of CP-I+Cortex (or BLA) (top), ACB-I+Cortex (or BLA) (middle), and ACB-I+CP-I+Cortex (or BLA) (bottom) projection motifs.

**(G)** Spatial expression of DEGs in (F) (Bregma: 2.1mm).


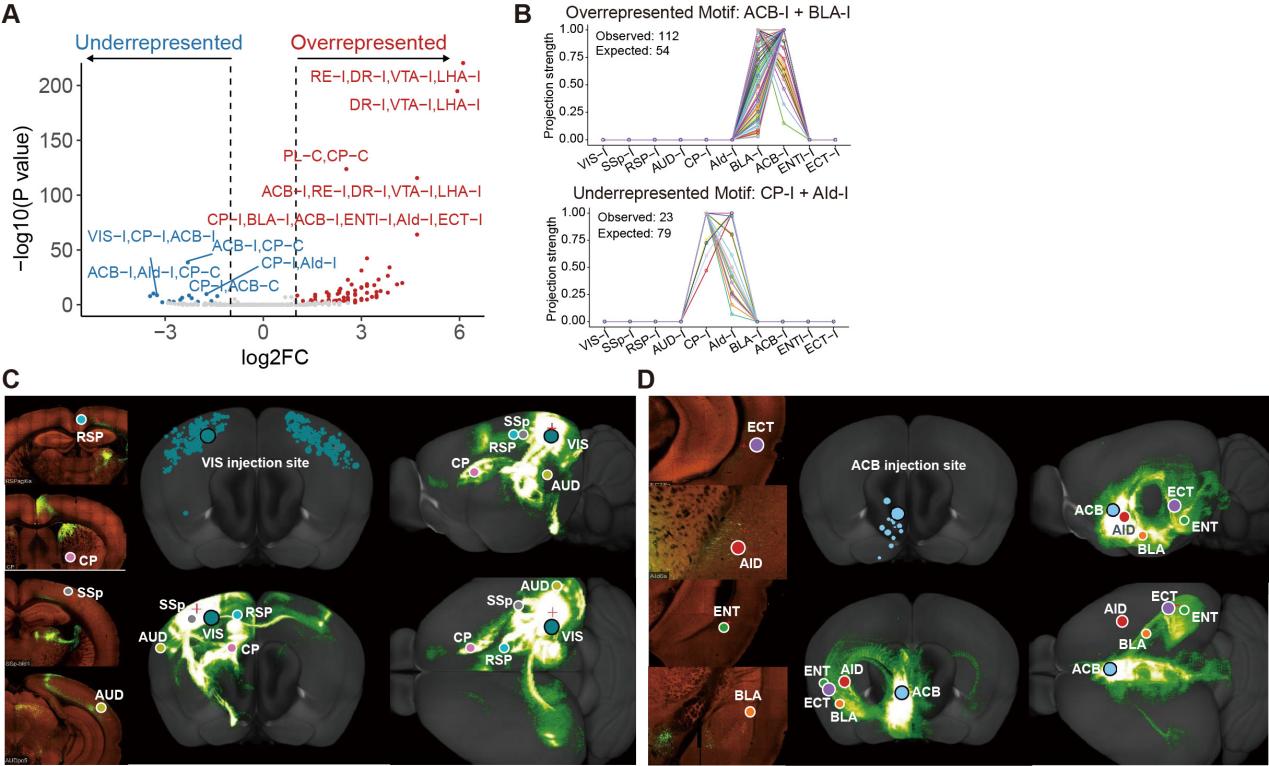


## Figure S10. Circuit connections between PFC downstream nuclei

(A) The volcano plot shows the under-represented and over-represented projection motifs compared to null model.

(B) Projection patterns of all individual neurons in the over-represented motif (ACB-I + BLA-I) and the under-represented motif (CP-I + AId-I).

(C) Circuit connections between downstream targets of ITi-M1. Data from the Allen Mouse Brain Connectivity Atlas.

(D) Circuit connections between downstream targets of ITi-M2. Data from the Allen Mouse Brain Connectivity Atlas.

**
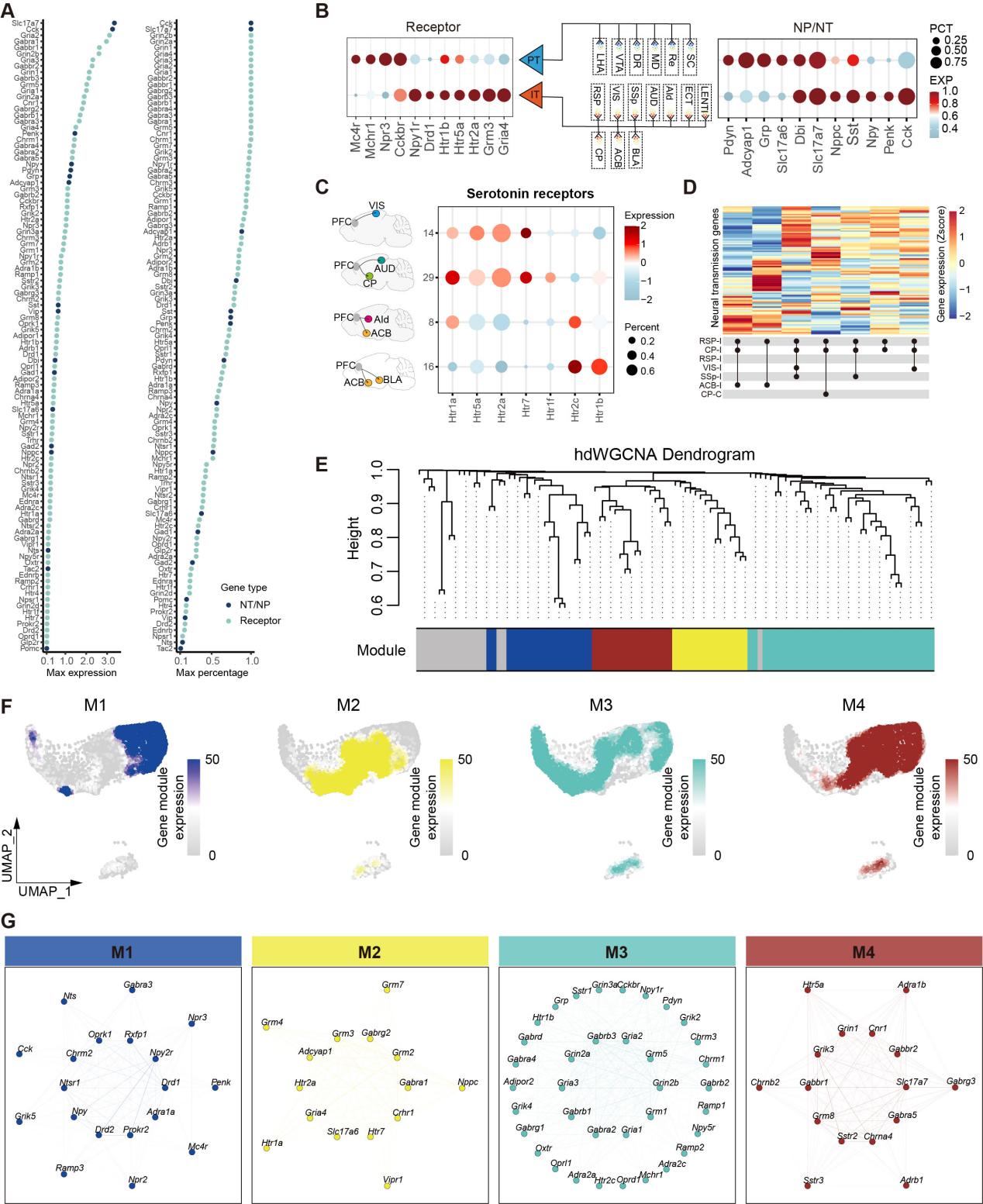
**

## Figure S11. Expression characteristics of neural signaling molecule

**(A)** The maximum expression levels (left) and cell percentage (right) of neural signal molecules (neurotransmitter (NT)/neuropeptide (NP) and receptor genes) in different PFC projection clusters.

**(B)** Differential neural signal transmission flow between IT and PT projection neurons of PFC. Dotplot showing the expression patterns of receptor genes (left) and neurotransmitter/neuropeptide genes (right) in IT/PT projection neurons. The middle panel showing the projection pattern of IT/PT projection neurons.

**(C)** Different projection clusters expressing diverse serotonin receptor subtypes.

**(D)** Different projection motifs target RSP-I have different neural signaling molecules expression patterns.

**(E)** hdWGCNA dendrogram of the co-expression network of molecules related to neuronal signaling molecules using hdWGCNA.

**(F)** UMAP colored by the module eigengenes (MEs) for the four gene modules.

**(G)** Co-expression plots for the four gene modules.


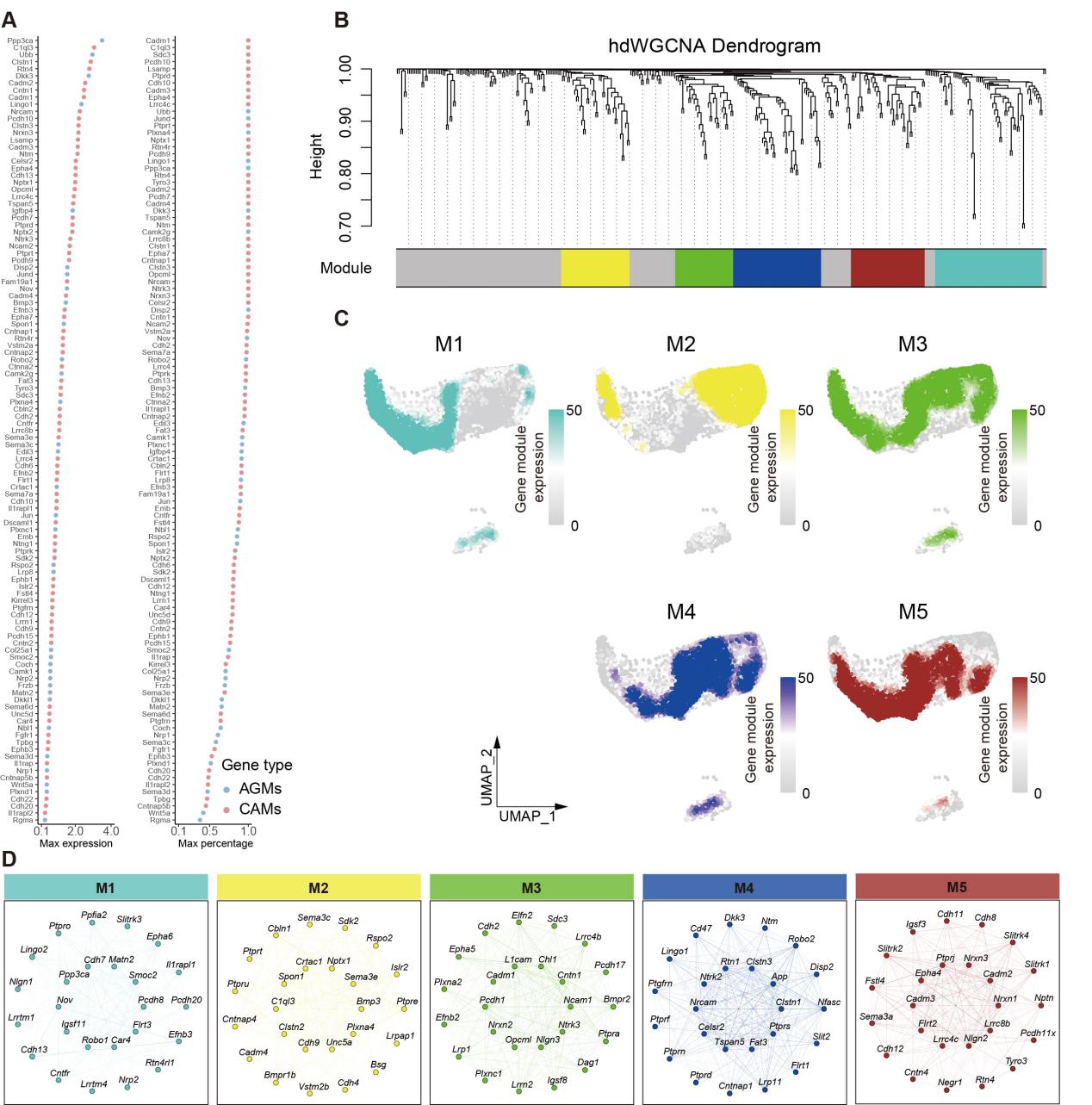


## Figure S12. Expression characteristics of neuronal circuit wiring related genes

**(A)** The maximum expression levels (left) and cell percentage (right) of axon guidance molecules (AGMs) and cadherin molecules (CAMs) genes in different projection neurons.

**(B)** hdWGCNA dendrogram of the co-expression network of molecules related to neuronal circuit wiring using hdWGCNA.

**(C)** UMAP colored by the module eigengenes (MEs) for the five gene modules.

**(D)** Co-expression plots for the five gene modules.

**
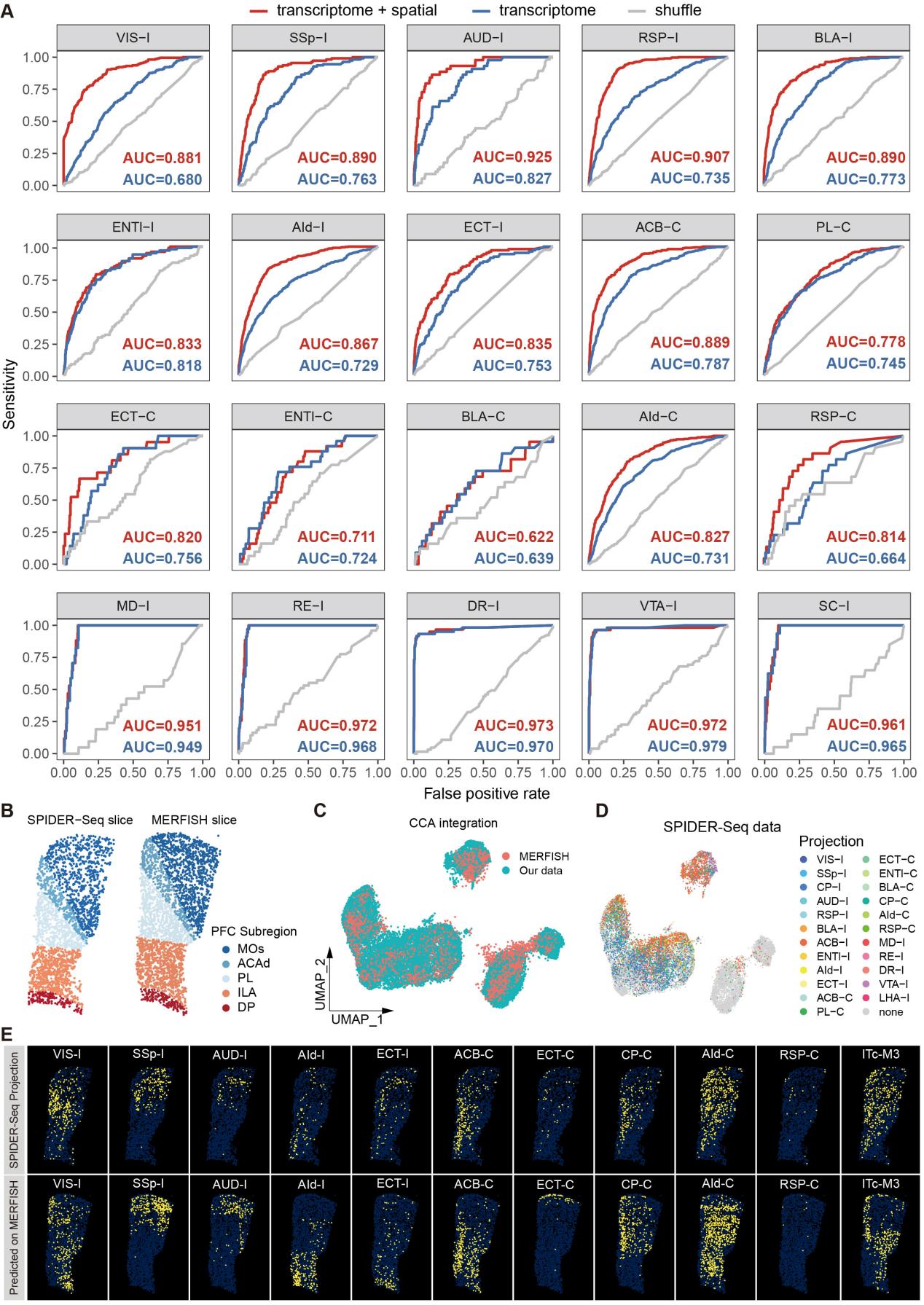
**

## Figure S13. Prediction of neuron projection by integrated gene profile and spatial location information by machine learning

**(A)** ROC curves for predicted projection targets. The red curves utilize both transcriptome and spatial information as input, the blue curves utilize only transcriptome as input, and the gray curves are random shuffle control.

**(B)** An example PFC slice from our SPIDER-Seq data (left) and the corresponding slice in MERFISH data (right), colored by PFC subregion.

**(C)** UMAP visualization of our SPIDER-Seq data and MERFISH data after CCA integration.

**(D)** UMAP visualization of our SPIDER-Seq data, with cells colored by the projection.

**(E)** The spatial distribution of neuron to different projection targets measured by SPIDER-seq (top), and the putative distributions of neuron to different targets predicted by our machine learning model based on MERFISH data (bottom) (Bregma: 1.78mm).

**
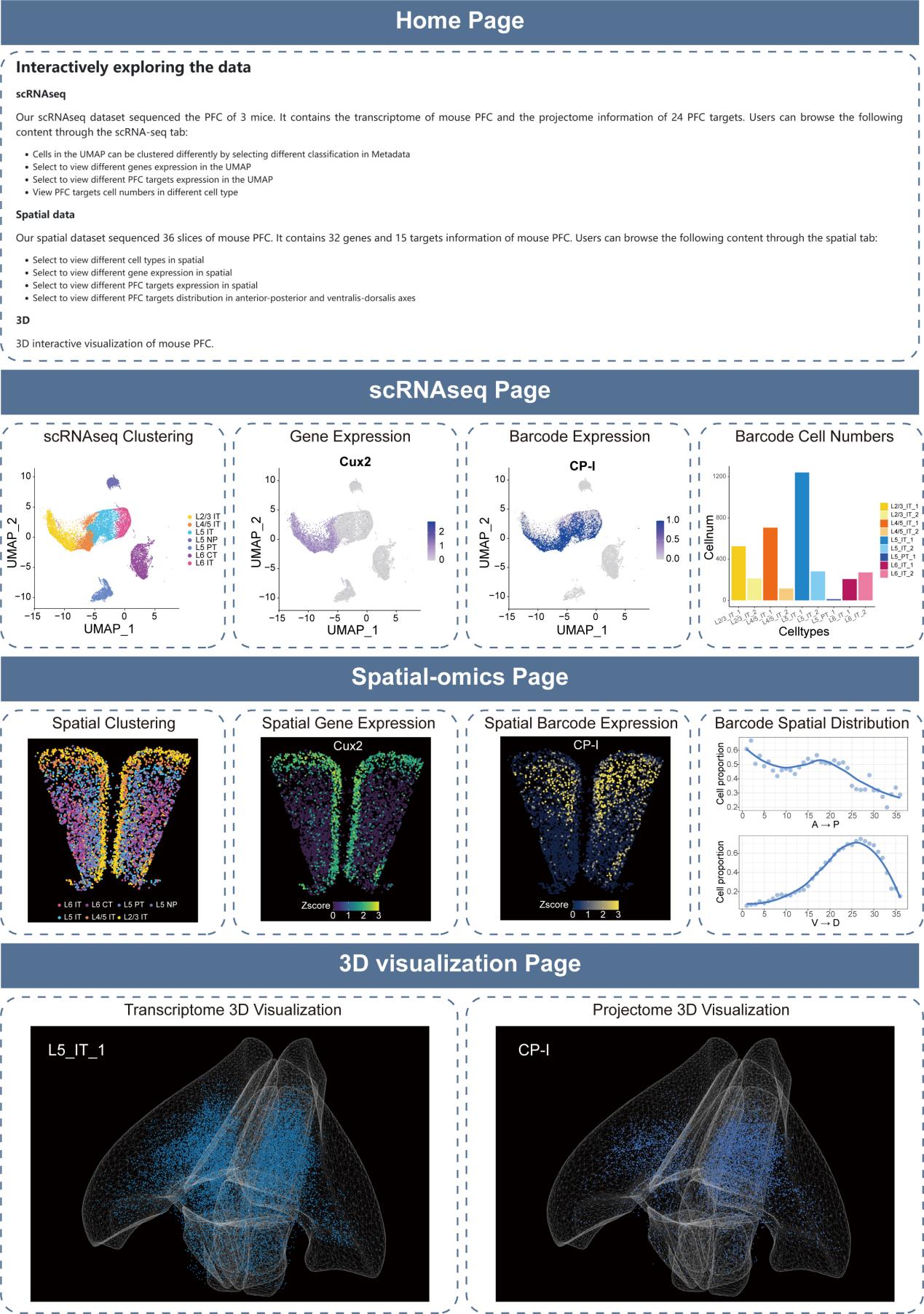
**

## Figure S14. Overview of the SPIDER-web

SPIDER-web is a shiny application that allows users to interactively access our data. Home Page provides information about our project and how to use it interactively. Users can access our scRNAseq data through scRNAseq Page, and access our spatial-omics data through Spatial Page. 3D visualization Page provides interactive 3D visualization of the PFC transcriptome and projectome.
