# Supplementary material for: Organization of mouse prefrontal cortex subnetwork revealed by spatial single-cell multi-omic analysis of SPIDER-Seq
Source: Natl Sci Rev. 2026 Jan 16;13(5):nwag004. doi: 10.1093/nsr/nwag004 (PMC12988354; doi:10.1093/nsr/nwag004)
Supplement: nwag004_Supplemental_Files [file nwag004_supplemental_files.zip › Supplementary_methods.docx]

SUPPLEMENTARY METHODS

Animal care

Animal care procedures and experiments (HZAUMO-2024-0318) were approved by the Scientific Ethics Committee of Huazhong Agricultural University, Hubei, China, and conducted ethically according to the Guide for the Care and Use of Laboratory Animals of the Research Ethics Committee of Huazhong Agricultural University. Mice were housed in facility with a standard light cycle (12 hr light/12 hr dark) and ad libitum access to food and water.

rAAV2-retro virus Barcoding

The rAAV2-retro barcode core plasmid (Plasmid #32395) was generated by replacing CMV-GFP-poly(A) with hSyn(452 nt)-GFP-BGH (225 nt), followed by insertion of NheI and BglII sequence downstream of the GFP. All barcode sequences were inserted between NheI and BglII by enzyme ligation or homologous recombination. We constructed 33 core plasmids carrying different barcode sequence (**Table S1**). The barcoding virus was rescued by transfecting the rAAV2-retro barcode plasmid, pAdDeltaF6 (Plasmid,112867) and rAAV2-retro helper plasmid (Plasmid,81070) into 293T cell line (ATCC,CRL-3216). The final product was purified by ioxanol ultracentrifugation to obtain the SPIDER-Seq tracing virus. The virus titer was adjusted to 2×10^12^ viral particles per mL.

Virus Injections

Female C57BL/6 mice at 7-8 weeks age were anaesthetized with a mixture of anesthetics 65 mg/kg ketamine and 13 mg/kg xylazine (i.p. injection). Their heads were fixed to a stereotaxic apparatus (68030, RWD, China). After exposed the skull, holes on the skull surface were drilled with corresponding coordinates (AP axis and ML axis, the coordinates for each nucleus are shown in **Table S2**). The virus was injected at a rate of 40 nl/μl, and 150 nl per nucleus. The micropipette was left in the tissue for 5 min before and after injection, to prevent virus spilling and backflow. The above operations were repeated to complete the injection of all nuclei (12-16 nucleus per mouse). The entire operation lasted about 6-8 h with 50 μl of anesthetic replenished every 3 hours. To compared the barcode in situ sequencing results with the viral tracing results of rAAV2-retro expressing fluorescent protein (EGFP or mCherry), we injected 150 nl of the rAAV2-retro expressing fluorescent protein into one target nucleus in each mouse. After surgery, the mice were placed on a heating pad to allow recovery and were allow housed under a 12-hour light/dark cycle at 22-25 °C for one month.

Single cell dissociation

One month after barcode virus injection, mice were anesthetized with isoflurane and decapitated. The brain was immediately removed and then sectioned into 300 μm slices in ice-cold ACSF (124 mM NaCl, 2.5 mM KCl, 1.2 mM NaH2PO4, 24 mM NaHCO3, 5 mM HEPES, 13 mM glucose, 2 mM MgSO4, and 2 mM CaCl2, pH: 7.3-7.4) on vibratome (Leica VT1200). Slices containing PFC region were transferred into Petri dish containing ice-cold ACSF with 45 µM Actinomycin D (Sigma-Aldrich, Cat# A1410). PFC region containing the anterior cingulate area (ACA), prelimbic area (PL), infralimbic area (ILA), secondary motor cortex (MOs), medial orbital cortex (ORBm), Dorsal peduncular area (DP) (∼Bregma 2.58 mm - Bregma 0.86 mm according to Paxinos and Franklin^,^s the Mouse Brain in Stereotaxic Coordinates) labelled with green fluorescence were isolated under a fluorescence microscope. Single cell suspensions were prepared as described previously [1]. The isolated tissues were quickly cut into small pieces less than 1 mm and transferred to digestion buffer containing 3 mg protease XXIII (Sigma-Aldrich, P5380) and 30 U/ml papain (Sigma-Aldrich, P3125). The digestion was performed at 34°C for 30 min and bubbled with a mixturegas of 95% O2 and 5% CO2 continuously. After the digestion, the tissue was transferred to stop buffer (ACSF contain 1 mg/ml Trypsin Inhibitor (Sigma-Aldrich, T6522), 2 mg/ml BSA (Sigma-Aldrich, A2153) and 1 mg/ml Ovomucoid Protease Inhibitor (Worthington, LK003153). We gently titrated the digested tissue with 4 polished Pasteur pipets, of which the bore diameter of the pipets is successively decreasing from 600 µm to 150 µm. Following trituration, suspension was filtered through a 30 mm filter, then centrifuged at 300 g for 5 minutes, The pellet was then resuspended in 700 µL ice-cold, carbogen-bubbled ACSF with 0.01% BSA and then subject to single cell RNA sequencing library preparation using BD Rhapsody single-cell Analysis System (BD Biosciences, 633702) according to the manufactory’s manual.

Single-cell RNA sequencing library preparation

Single-cell mRNA sequencing library was performed by the BD Rhapsody Single-Cell Analysis System (BD Biosciences, 633702). Firstly, single-cell suspension in BSA was loaded into a BD Rhapsody cartridge (BD Biosciences, 633733) with >200,000 microwells. Secondly, single-cell mRNA was captured by magnetic beads with barcoded capture oligos (BD Biosciences, 664887). Then, magnetic beads were collected for cDNA synthesis and library construction following the BD Rhapsody single cell 3' whole transcriptome amplification (BD Biosciences, 633733) workflow. Finally, the libraries were sequenced on Illumina NovaSeq 6000 (Illumina, USA) with 300-bp reads (150-bp paired-end reads).

*In situ* Sequencing of the transcripts of PFC genes and barcodes

Probe design: We modified the protocol according to previous in situ sequencing method [2]. Briefly, 4-20 pairs of probes were designed for each gene depending on the length of mRNA (marker gene or barcode), and all probe sequences were shown in **Table S3**. Each end of the padlock probe contains 13 nt complementary to the target sequence, and the middle region contains two repeats of the complementary sequence of the detection probe. The 5' and 3' end of initiator primer are complementary to the 3' end of the targeting sequence and padlock probe, respectively. We customized 12 groups of 16 bp fluorophore-modified detection probes (Alexa Fluor 488, CY3, CY5, and CY7) (**Table S4**). 47 gene are detected by 12 imaging cycle and five channels for DAPI, 488, cy3, cy5, cy7 are used in each cycle to rearch more robust and efficient results. The padlock probe was phosphorylated with T4 Polynucleotide Kinase at 200 µM (Vazyme, N102-01), and then annealed with initiator primer. Detection probes were dissolved at 100 µM in ultrapure RNase-free water. All probes were stored at -80°C before use.

Slices preparation: One month after virus injection, the mice were perfused with 4% PFA, and postfixed in 4% PFA for 24 h. We then cryoprotected the brain with 30% sucrose until tissue sinks and embed the mice brain in OCT. The brain tissue was sliced to 15 µm continuously with a Leica cryostat (Leica CM3050 S). Slices containing PFC were mounted on the poly-L-lysine pretreated cover glass then stored at -80°C until used. The brain slices were sealed in Secure-Seal hybridization chambers (Grace, 621505), fixed with 4% PFA for 10 mins, permeabilized with pre-cooled methanol at -80°C for 15min, then followed by pepsin (2 mg/mL, Sigma, P0525000) digestion at 37°C for 90 s.

The probes were diluted at 25 µM to 100 µM in hybridization buffer with 2X SSC (Sangon, B548109) ,10% formamide (Sangon, A100606) and 20 mM RVC (Beyotime, R0108)) and incubated overnight at 37°C. The brain slices were washed twice in PBSTR (0.1% Tween-20, 0.1 U/µL RRI in PBS) for 20 min, followed by wash in 4X SSC dissolved in PBSTR. Next, the brain slices were incubated in the ligase reaction mixture (1 U/µL SplintR ligase (NEB, M0375L), 1X buffer, 0.2U/µL RRI) for 2 hours at 25°C. Following 20 mins wash with PBSTR, and then incubated with RCA mixture (1 U/µL Phi29 (Vazyme, N106-01), 1X RCA buffer, 0.25 µM dNTP, 0.2 µg/µL BSA, 5% Glyceryl)) at 30°C for 6 hours.

Imaging: Slices were incubated with detection probe labelled with fluorescence (488, cy3, cy5, cy7) at 37°C for 30min in each round, and washed with PBST for 3 times, following with DAPI staining. Slice were scanned by Leica THUNDER Imager system with 20× lens (NA 0.75). After each round of imaging, signals were stripped by stripping buffer (60% formamide in 2X SSC) twice at room temperature for 10 mins for each round. DAPI staining was performed 3 cycle of image.

scRNA-seq and barcode data pre-processing

Raw reads were pre-processed using the BD Rhapsody™ Whole Transcriptome Analysis (WTA) pipeline (v1.11) (<https://bd-rhapsody-bioinfo-docs.genomics.bd.com>). The R1 reads were analyzed to identify the cell label sequences (CLS), common linker sequences (L), and Unique Molecular Identifier (UMI) sequence. The R2 reads were used for aligning to the reference genome and annotating genes. For WTA reference genome, we selected GRCm38-PhiX-gencodevM19-20181206.tar file. For transcriptome annotation, we selected gencodevM19-20181206.gtf file. After setting up, we ran pipeline using the default parameters. The expression matrix file generated by pipeline was used for transcriptome analysis, while the BAM file was used for extracting projectome information.

scRNA-seq quality control

Single cell RNA-seq transcriptome analysis is mainly performed by R package Seurat (v4.4.0) [3]. Briefly, Seurat object was created using the “CreateSeuratObject” function, and the gene expression profile of each cell was then normalized using the “NormalizeData” function with scale.factor = 10000. We filtered the following cells: nCount_RNA < 1000, nFeature_RNA < 1000, and mitochondrial contents > 15%. We removed neuronal cells with nFeature_RNA < 1500, because previous studies showed that neuronal cells have a higher number of genes expressed than non-neuronal cells [4]. The following genes were filtered: min.cells < 3, mitochondrial genes, and ribosomal genes. Then, the R package DoubletFinder (v2.0.3) [5] was used to remove potential doublets.

Clustering of scRNA-seq transcriptome

First Seurat Canonical Correlation Analysis (CCA) was used to remove batch effects between three samples. The “SelectIntegrationFeatures” function was applied to select top 2000 common variable genes across three samples. Then we used the “FindIntegrationAnchors” function to find anchors, and integrated the three datasets together using the “IntegrateData” function. After integration, we performed the standard Seurat clustering analysis workflow. We used the “ScaleData” function to scale the integrated data, and performed principal component analysis (PCA) using the “RunPCA” function. Then we computed the nearest neighbors used the “FindNeighbors” function with top 20 PCs. We used the “FindClusters” function for clustering analysis with resolution = 0.5. Then, we annotated the clusters based on previously reported markers of PFC cell types [6]. We manually removed some mixed low-quality cell clusters that expressed markers of multiple cell types. Seven main cell types were annotated: Excitatory neuron, Inhibitory neuron, Astro, Endo, Microglia, Oligo, OPC. Then, we extracted Excitatory cell type for further analysis. We used the “FindClusters” function for Excitatory clustering analysis with resolution = 2. Then, we annotated the clusters based on previously reported markers of PFC Excitatory subtypes. We annotated a total of 13 Excitatory subtypes: L2/3_IT_1, L2/3_IT_2, L4/5_IT_1, L4/5_IT_2, L5_IT_1, L5_IT_2, L6_IT_1, L6_IT_2, L5_PT_1, L5_PT_2. Then, we ran the Uniform Manifold Approximation and Projection (UMAP) dimensional reduction using the “RunUMAP” functions, and visualized data using functions provided by Seurat. In total, our data contains a transcriptome expression matrix of 33,766 cells and 26,902 genes. 9,038 neurons were labeled with barcodes (1,842 for mouse1; 4,410 for mouse2; 2,786 for mouse3).

Projectome barcode alignment

We constructed a ‘database.fasta’ file containing our barcode sequences, and used the blast makeblastdb command (v2.13.0) to produce BLAST databases. Then, we used the samtools (v1.13) [7] software to extract R2 reads sequences, cell labels, and Unique Molecular Identifier (UMI) sequences from the BAM file, and constructed a ‘query.fasta’ file. We used the blastn command to align the ‘query.fasta’ file with the barcode BLAST database using the following parameters: -task blastn-short -word_size 4 -evalue 1 -outfmt "6 qseqid sseqid nident" -max_hsps 1 -ungapped -num_threads 10 -mt_mode 1. We kept sequences with 3 or fewer mismatches and removed sequences with duplicate UMI to generate the cell-barcode projectome expression matrix.

Removing barcodes background noise

We observed low expression of barcodes in some non-neuronal cells in the projectome expression matrix. Due to the slightly different sequencing depths of the 3 scRNAseq samples, we set different thresholds for different samples to filter out background noise accordingly. First, we divided the cells into Neuron and Non-neuron categories and plotted the UMI Counts-Barcode curve. We used the UMI Counts of the elbow point position of the Non-neuron curve as the threshold, and set the barcode expression below the threshold in Neuron and Non-neuron as 0. Finally, to compare the projection patterns between neurons on the same scale, we performed Min-Max Normalization on the expression levels of barcodes in each cell. With that, the raw barcode counts were rescaled between zero and one.

Clustering of single cell projectome

R stats (v4.2.0) "hclust" function was used to perform hierarchical cluster analysis for the cell-barcode matrix. The "cutree" function was then applied to cut the tree into clusters at a height=1.5. After manually merged clusters with similar barcode expression profiles, we obtained a total of 33 projectome clusters. Then, we run UMAP dimensional reduction on the cell-barcode matrix, and merged 33 projectome clusters into 4 projectome modules.

Visualization of the distribution of projection neurons on UMAP

Density scatter plots were used to visualize the distribution of different projection neurons on the UMAP. Specifically, we used the "geom_pointdensity" function from R package ggpointdensity (v0.1.0) to create scatterplots where each point was colored by the number of neighboring points. This is useful to visualize the 2D-distribution of points in case of overplotting.

Calculation of projectome correlation between SPIDER-Seq and fMOST data

To verify the reproducibility of SPIDER-Seq projectome data, we collected fMOST projectome data from Gao et al. [8]. It contains the number of neurons that PFC project to downstream targets (**Tables S6**). We compared the number of neurons project to each target with our SPIDER-Seq data. We plotted the correlation curves and calculated the Pearson correlation coefficient (**Fig. S1O**). Each point represents a target, the X and Y axes represent the projection intensity in fMOST and SPIDER-seq (Min-Max normalization). The correlation between SPIDER-seq and fMOST projections reached 0.81.

Calculation of projection motifs

To calculate the statistically significant projection motifs, we constructed a null model based on a previous study [9]. Briefly, we first assumed that each neuron projected to each target were independent, and used the binomial cumulative distribution function to calculate the expected cell number of each projection motifs. We then calculated the P value by comparing the expected cell number to the observed cell number, and corrected the P value with the Bonferroni method. We defined the projection motifs with log2 fold change > 1 or < -1 and P value < 0.01 as significantly over- or underrepresented projection motifs.

Calculation of the correlation between projectome, spatial location, and transcriptome

The spatial, transcriptome, and projectome distances for different projection motifs were calculated to plot the correlation scatter plot. Specifically, we used the “dist” function in R to calculate the euclidean distance between each pair of projection motifs. The "geom_pointdensity" function from R package ggpointdensity (v0.1.0) was used to create the scatterplots. Correlation was calculated using the Pearson correlation coefficient.

Image registration and cell segmentation

The raw images were registered for each round using BigWarp (v9.1.2). Specifically, we used the first round image as the reference and manually aligned the corresponding manually labelled“aligning points” between each round image with the first round image in BigWarp's "landmark mode". After alignment, we performed image registration. We used FIJI to stack all channels and rounds of images of the same slice into one image file. We used the DAPI channel of each slice to perform cell segmentation by Cellpose (v2.0.5) [10] and then calculated the average gray value of each channel in each cell to generate the spatial cell-channel expression matrix.

Spatial expression matrix quality control

We manually set thresholds for quality control on the spatial cell-channel expression matrix to filter out values with low channel expression to remove background noise. We filtered out cells with a volume smaller than 50 pixels (32.5um^2^) or larger than 500 pixels (325um^2^) in each slice, as well as the cells with total expression levels less than 5 or higher than 400, to remove low-quality cells and potential doublets. Then, we loaded the expression matrix into Seurat and used the "NormalizeData" function for normalization, with the parameter scale.factor=100.

Spatial cell annotation

To annotate cells in the spatial data, we used Tangram (v1.0.4) [11] to map the annotated information of scRNAseq clusters onto the cells in the spatial-omics map. Briefly, we used “tg.pp_adatas” function to find the common genes between adata_sc and adata_sp. Then we used the "tg.map_cells_to_space" function to perform the cluster level mapping, with parameters: mode='clusters', cluster_label='SubType'.

PFC 3D visualization

For 3D visualization of PFC, we used R package WholeBrain (v0.1.1) [12] and extended functions [6] to align the spatial slices with Allen Brain Atlas CCF v3. First, each brain slice was paired to the closest matching coronal section in CCF v3 with the help of DAPI image and spatial location of the cell types. Then, we manually adjusted the scale and position of each brain slice to ensure accurate alignment with CCF v3. After alignment, we extracted the PFC regions according to the brain region annotation information in CCF v3. Then, we used R package rgl (v1.1.3) and Allen brain 3D mesh [13] to visualize PFC in 3D.

Single cell weighted gene co-expression network analysis

Single cell weighted gene co-expression network analysis is mainly performed by R package hdWGCNA (v0.3.01) [14]. Briefly, "SetupForWGCNA" function was used to select neural signal molecule or neural circuit wiring molecule genes for analysis. We used "MetacellsByGroups" function to construct metacell expression matrix, and normalized the matrix using "NormalizeMetacells" function. Then, "SetDatExpr" function was used to specify the expression matrix for network analysis, and soft power threshold was selected using "TestSoftPowers" function. We used "ConstructNetwork" function to construct the co-expression network, and visualized the network using functions provided by hdWGCNA. Module Eigengenes (MEs) are a commonly used metric to summarize the gene expression profile of an entire co-expression module. Briefly, module eigengenes are computed by performing principal component analysis (PCA) on the subset of the gene expression matrix comprising each module. The first PC of each of these PCA matrices are the MEs. We used the "ModuleEigengenes" function from R package hdWGCNA (v0.3.0.1) to calculate the MEs for each gene co-expression module. To fit the correlation curve, we divided cells into 10 bins, ranging from 10% to 100%, based on the MEs values. We then calculated the average projection intensity and MEs for each bin and plotted the correlation curve.

Neuron projection prediction by machine learning

We trained a XGBoost model to predict projectome by transcriptome and spatial location in each neuron. In order to simultaneously obtain the whole transcriptome and spatial location of single neuron, we calculated the projectome correlation coefficient between each cell in scRNAseq dataset and spatial-omics dataset according to their embedded projection information in both datasets. Based on the projection similarity, the spatial location (X, Y, Z) of a neuron in the spatial-omics dataset was assigned to the neuron in scRNAseq dataset with the highest projection correlation coefficient. We used the R package caret (v6.0-94) “createDataPartition” function to split the scRNAseq cells into training and test datasets with a 7:3 ratio. The first 30 PCs of the transcriptome and the spatial X, Y, and Z coordinates were used as features, and the binary annotation of the projection module or target area were used as the labels. We first used the R package xgboost (v1.7.5.1) "xgb.cv" function to find the optimal nround, and then used the "xgboost" function to train model, using the following parameters: max_depth=5, eta=0.5, nthread = 5, nround = xgb.cv$best_iteration, objective = "binary:logistic". Next, we used the "predict" function to make predictions on the test dataset. ROC curve and area under the curve (AUC) values were obtained using R package PRROC (v1.3.1) "roc.curve" function. To further verify the generalization ability of our XGBoost model, we performed projectome predictions based on PFC MERFISH data from previous a study [6]. We first integrated the MERFISH data with SPIDER-Seq data using Seurat's standard CCA analysis pipeline. Then the first 30 PCs of the transcriptome and the X and Y coordinates were used as features. SPIDER-Seq data were used as the training data and the PFC MERFISH data were used as the test data.

Reference

1. Saunders A, Macosko EZ, Wysoker A *et al.* Molecular Diversity and Specializations among the Cells of the Adult Mouse Brain. *Cell*. 2018; **174**(4): 1015-1030.e1016. doi: 10.1016/j.cell.2018.07.028

2. Wu X, Xu W, Deng L *et al.* Spatial multi-omics at subcellular resolution via high-throughput in situ pairwise sequencing. *Nature biomedical engineering*. 2024; **8**(7): 872-889. doi: 10.1038/s41551-024-01205-7

3. Hao Y, Hao S, Andersen-Nissen E *et al.* Integrated analysis of multimodal single-cell data. *Cell*. 2021; **184**(13): 3573-3587.e3529. doi: 10.1016/j.cell.2021.04.048

4. Bhattacherjee A, Djekidel MN, Chen R *et al.* Cell type-specific transcriptional programs in mouse prefrontal cortex during adolescence and addiction. *Nature communications*. 2019; **10**(1): 4169. doi: 10.1038/s41467-019-12054-3

5. McGinnis CS, Murrow LM, Gartner ZJ. DoubletFinder: Doublet Detection in Single-Cell RNA Sequencing Data Using Artificial Nearest Neighbors. *Cell systems*. 2019; **8**(4): 329-337.e324. doi: 10.1016/j.cels.2019.03.003

6. Bhattacherjee A, Zhang C, Watson BR *et al.* Spatial transcriptomics reveals the distinct organization of mouse prefrontal cortex and neuronal subtypes regulating chronic pain. *Nature neuroscience*. 2023; **26**(11): 1880-1893. doi: 10.1038/s41593-023-01455-9

7. Danecek P, Bonfield JK, Liddle J *et al.* Twelve years of SAMtools and BCFtools. *GigaScience*. 2021; **10**(2). doi: 10.1093/gigascience/giab008

8. Gao L, Liu S, Gou L *et al.* Single-neuron projectome of mouse prefrontal cortex. *Nature neuroscience*. 2022; **25**(4): 515-529. doi: 10.1038/s41593-022-01041-5

9. Han Y, Kebschull JM, Campbell RAA *et al.* The logic of single-cell projections from visual cortex. *Nature*. 2018; **556**(7699): 51-56. doi: 10.1038/nature26159

10. Stringer C, Wang T, Michaelos M *et al.* Cellpose: a generalist algorithm for cellular segmentation. *Nature methods*. 2021; **18**(1): 100-106. doi: 10.1038/s41592-020-01018-x

11. Biancalani T, Scalia G, Buffoni L *et al.* Deep learning and alignment of spatially resolved single-cell transcriptomes with Tangram. *Nature methods*. 2021; **18**(11): 1352-1362. doi: 10.1038/s41592-021-01264-7

12. Fürth D, Vaissière T, Tzortzi O *et al.* An interactive framework for whole-brain maps at cellular resolution. *Nature neuroscience*. 2018; **21**(1): 139-149. doi: 10.1038/s41593-017-0027-7

13. Ortiz C, Navarro JF, Jurek A *et al.* Molecular atlas of the adult mouse brain. *Science advances*. 2020; **6**(26): eabb3446. doi: 10.1126/sciadv.abb3446

14. Morabito S, Reese F, Rahimzadeh N *et al.* hdWGCNA identifies co-expression networks in high-dimensional transcriptomics data. *Cell reports methods*. 2023; **3**(6): 100498. doi: 10.1016/j.crmeth.2023.100498
